# Spontaneous chiral symmetry breaking in a single crystal

**DOI:** 10.1039/d5sc02623g

**Published:** 2025-08-19

**Authors:** Ryusei Oketani, Musashi Okada, Kentaro Takaji, Hajime Shigemitsu, Toshiyuki Kida, Takuya Nakashima, Ichiro Hisaki

**Affiliations:** a Division of Chemistry, Graduate School of Engineering Science, The University of Osaka 1-3 Machikaneyama Toyonaka Osaka 560-8531 Japan r.oketani.es@osaka-u.ac.jp i.hisaki.es@osaka-u.ac.jp; b Department of Applied Chemistry, Graduate School of Engineering, The University of Osaka 2-1 Yamadaoka Suita Osaka 565-0871 Japan; c Department of Chemistry, Graduate School of Science, Osaka Metropolitan University 3-3-138 Sugimoto Sumiyoshi Osaka 558-8585 Japan

## Abstract

Chiral symmetry breaking (CSB) under nonequilibrium open conditions is a ubiquitous phenomenon in the universe, whereas molecular-level CSB is limited. Only the preferential enrichment and Viedma ripening that occur during the crystallization process from a solution are known. Herein, we discovered the third category of CSB, which is complete CSB within a single crystal. A racemic crystal of 3-(4-(benzo[*d*]thiazol-2-yl)phenyl)-10-propyl-10*H*-phenothiazine, a phenothiazine derivative with dynamic chirality, undergoes a single-crystal-to-single-crystal structural transition to a chiral crystal. Furthermore, the chirality after the transition is able to be controlled by solid-seeding of a chiral crystal. The resulting chiral single crystals exhibited circularly polarized luminescence (CPL) properties (*g*_lum_ = 8.9 × 10^−4^). This discovery provides a simple model of CSB and stimuli-responsive materials involving the CSB phenomenon.

## Abstract 2

We discovered SC–SC transition from achiral to chiral crystal accompanying chiral symmetry breaking (CSB). This process would provide the simple CSB model for understanding molecular-level CSB.

## Graphical abstract

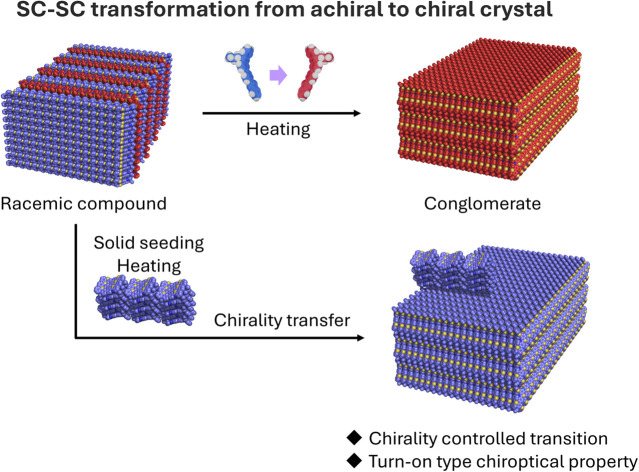

## Introduction

Spontaneous chiral symmetry breaking (CSB) is a crucial phenomenon in the formation of the universe that has long fascinated scientists.^1,2^ The discovery of molecular chirality by Louis Pasteur^3^ and the overwhelming prevalence of l-amino acids in biological systems have captivated researchers.^4,5^ While the origin of homochirality remains unresolved, most proposed mechanisms involve the presence of external chiral environments, such as template surfaces^6–8^ and polarized light.^9–12^ Fundamentally, spontaneous CSB refers to processes in which a metastable racemic system becomes a stable chiral system and can be distinguished from the chirality amplification induced by chiral dopants or other external factors.

**Fig. 1:**
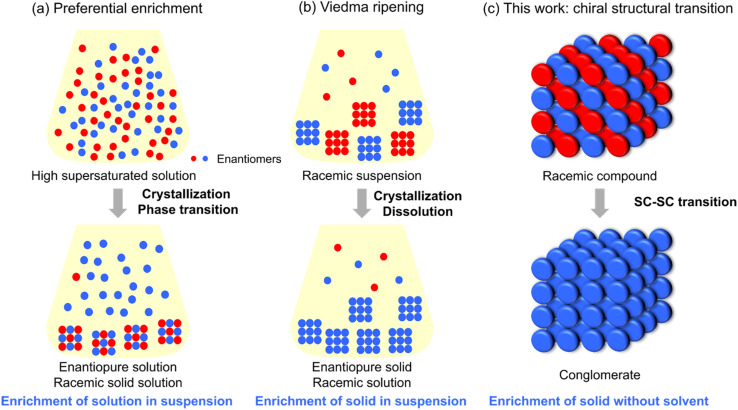
CSB phenomena at the molecular level. (a) Preferential enrichment, (b) Viedma ripening, and (c) structural transition from an achiral crystal to a chiral crystal.

At the molecular level, two CSB phenomena in nonequilibrium open systems are known: preferential enrichment^13^ and Viedma ripening.^14,15^ Preferential enrichment is a symmetry breaking and chiral amplification process triggered by polymorphic transitions during the crystallization of racemic compounds from supersaturated solutions.^16,17^ The deposited crystals are nearly racemic, while the resulting solution becomes highly enriched (Fig. 1a). Applications of this method to amino acid derivatives and pharmaceutical compounds have been reported.^18^ Viedma ripening, in contrast, was observed in CSB occurring during sodium chlorate crystallization. Although Kondepudi and coworkers reported this CSB first,^19^ enrichment from the racemic suspension was proved by Viedma.^20^ When the conditions for racemization of constituents in solution and crystallization as a conglomerate are met simultaneously, the crystal phase in a racemic suspension is known to be enriched in one enantiomer (Fig. 1b). Over the past two decades, extensive research on Viedma ripening has been conducted, including its application to various organic molecules,^21–24^ its combination with reactive crystallization,^25–27^ and its process engineering^28–31^ involving temperature-cycle induced methods^32,33^ and model simulations.^34,35^

These CSB processes have significant potential in pharmaceutical and materials chemistry. They are being explored as potential resolution methods and are expected to serve as fundamental tools in these fields. In materials chemistry, chiral structures are particularly valuable for applications in circularly polarized luminescent materials,^36–39^ chiral recognition materials,^40–42^ and nonlinear optical materials.^43,44^ Even compounds that are achiral in solution can form chiral structures when they crystallize in space groups belonging to the Sohncke group. This result implies that achiral compounds should be included in the search space for chiral materials, which necessitates methods to obtain enantiopure solids. While Viedma ripening yields products in crystalline form, preferential enrichment increases enantiomeric excess of the solution phase and is thus inapplicable to achiral compounds. In either case, a definitive purification method for obtaining chiral crystals of achiral compounds has yet to be established.

To understand CSB, simple systems exhibiting this phenomenon must be discovered. While CSB can be understood within the thermodynamic framework of nonequilibrium open systems,^45,46^ there is no existing theory that can predict compounds capable of inducing this phenomenon. Specific phenomena can be understood through individual case studies, but this approach is unlikely to significantly contribute to a general understanding of molecular-level CSB. The challenges in this field can be attributed to the fact that there are only two known examples of molecular-level CSB, which occur in complex systems involving solutions. Consequently, to model the entire system, multiple types of molecules at different scales must be considered, *i.e.*, numerous solvent molecules and a few solute molecules to simulate a precise system. To explore applications in other systems, simple systems must be discovered and thoroughly understood.

Herein, we report a groundbreaking discovery: the third category of molecular-level CSB. The thermodynamically metastable racemic compound of a chiral phenothiazine derivative, 3-(4-(benzo[*d*]thiazol-2-yl)phenyl)-10-propyl-10*H*-phenothiazine (1), underwent a single-crystal-to-single-crystal (SC–SC) structural transformation into a stable conglomerate upon heating (Fig. 1c). Although the molecule possesses point chirality at the nitrogen atom of the phenothiazine core, it is not strictly chiral in solution because rapid racemization occurs at room temperature. Remarkably, the chirality of one of the two enantiomers in the original racemic compound was inverted within the crystal, resulting in convergence to a single chirality. This phenomenon occurred in an ultimately simple system without the intervention of solution-phase molecules. The chirality transfer from a conglomerate with a predetermined chirality to a racemic compound upon the transition was confirmed. This result demonstrates that the chirality after the transition can be induced by solid-seeding. Furthermore, we discovered that the post-transition crystal exhibited circularly polarized luminescence (CPL) characteristics with *g*_lum_ = 8.9 × 10^−4^. Through this SC–SC structural transformation from a racemic compound to a conglomerate, we realized a turn-on type CPL-emitting material.

## Results

### Selective preparation of chiral and achiral crystals and their crystal structures

**Fig. 2:**
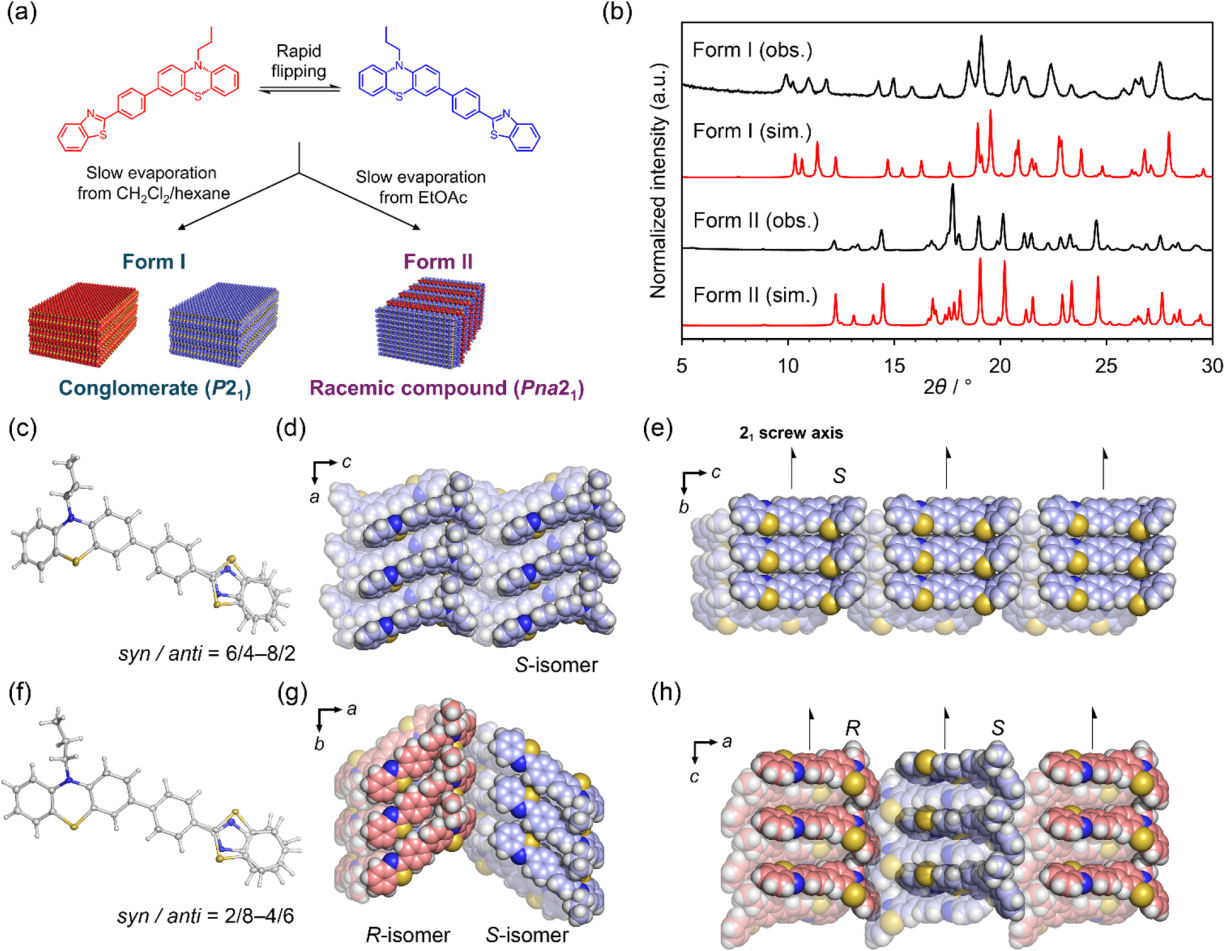
(a) Selective preparation conditions for forms I and II from solution. (b) PXRD patterns for form I grown from EtOAc and form II grown from CH_2_Cl_2_/heptane (black lines), and simulated patterns for forms I and II (red lines). Crystal structures of (c–e) form I and (f–h) form II. Anisotropic displacement ellipsoid plots of (c) form I and (f) form II are drawn with 50% probability. The benzothiazole moiety was disordered into *syn* and *anti* forms, referring to the relative positions of the sulphur atoms on the phenothiazine and benzothiazole. Downward views of the packing structures along the 2_1_ screw axes for (d) form I and (g) form II. The space filling models in red and blue indicate the *R* and *S* isomers, respectively. The side views from the 2_1_ screw axes for (e) form I and (h) form II.

Ekbote and coworkers reported that chiral and achiral crystals of 1 were concomitantly grown from a CH_2_Cl_2_ solution.^47^ In contrast, we found the selective preparation conditions of chiral and achiral crystals and the details of disordered structures including occupancy ratios. The chiral crystal, form I, was selectively prepared by slow evaporation from tetrahydrofuran (THF), benzene, *tert*-butyl methyl ether (MTBE), diethyl ether, CH_2_Cl_2_/heptane, and CHCl_3_/heptane solutions (Fig. 2a). Form I crystals typically have needle- or block-shapes. The achiral crystal, form II, was selectively prepared by slow evaporation from ethyl acetate. Form II crystals have plate shapes. The selective preparation of each crystal structure was confirmed using powder X-ray diffraction (PXRD) patterns and simulated patterns from single crystal X-ray diffraction (SCXRD) data (Fig. 2b).

**Fig. 3:**
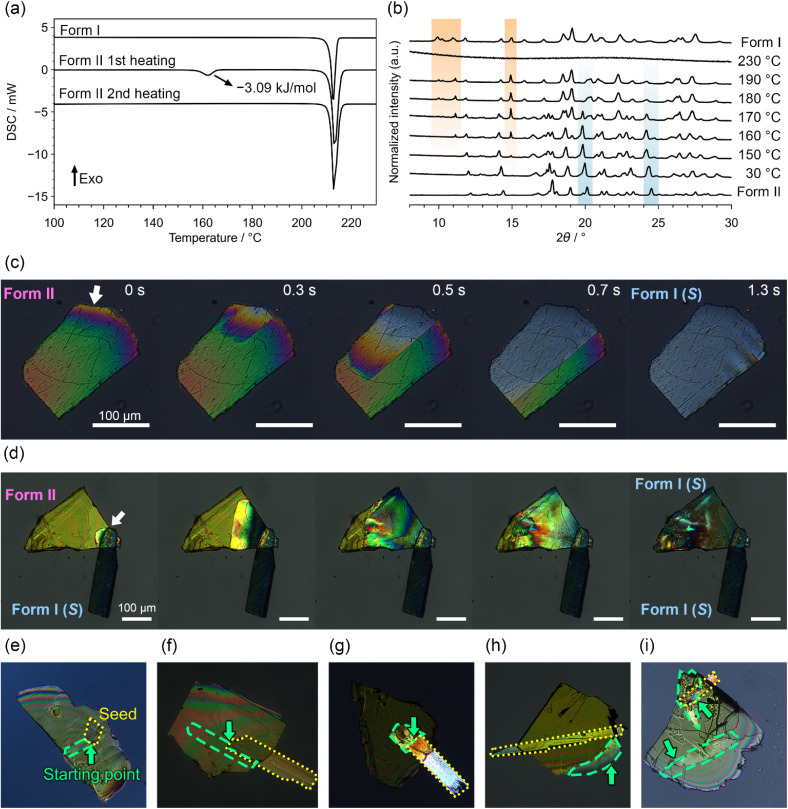
(a) DSC curves for forms I and II. The thermograms for form I and the second heating of form II are plotted with a vertical offset of 4 mW for clarity. The heating rate was 5 K min^−1^. (b) VT-PXRD patterns for form II. The observed patterns for forms I and II are indicated. The orange and blue shaded regions indicate the characteristic peaks for forms I and II, respectively. (c) POM images during the SC–SC structural transformation from form II to I at 180 °C. The scale bar represents 100 μm. (d) POM images during chirality transfer from form I to II by solid-seeding at 150 °C. (e–g) POM images of typical progression behaviour when the transition started from the contact point. Areas with yellow frames indicate a seed crystal. The arrows indicate the starting point of the transition. Areas with green frames indicate where the transition occurred. (h) POM image during the transition initiated from the edge of the crystal. (i) POM image during the transition initiated from multiple points.

Ekbote and coworkers also reported the crystal structures of these forms; however, we noticed the disordered structure of benzothiazole that was not mentioned in the previous paper during the redetermination of the crystal structures (Table S2). Form I was a crystal structure with space group *P*2_1_, and the benzothiazole moiety was observed as a disordered structure with an inverted site, called *syn* or *anti* based on the relative position of the sulphur atoms (*a* = 8.5534(3) Å, *b* = 5.5300(2) Å, *c* = 22.8598(7) Å, *β* = 94.991(3)° at 113 K, *R*_1_ = 5.35%, and *wR*_2_ = 18.81%) (Fig. 2c–e). The *syn*/*anti* occupancy slightly varied among the crystals, but generally ranged from 6/4 to 8/2. The racemic compound phase had a crystal structure with the space group *Pna*2_1_, and a similar disordered structure was observed (*a* = 39.5906(7) Å, *b* = 7.17130(10) Å, *c* = 7.64230(10) Å, *α*, *β*, *γ* = 90° at 113 K, *R*_1_ = 4.23%, and *wR*_2_ = 9.62%) (Fig. 3f–h). Similar to form I, the *syn*/*anti* ratio slightly varied among the crystals, ranging from 2/8 to 4/6. Both crystal structures had a 2_1_-screw axis along the short axis of the molecule. Differences between the two forms were found in the conformation of the molecules. The bending angle of the phenothiazine ring *θ*_1_ was 135.57° in form I and 149.58° in form II (Fig. S2 and Table S6). The angle between phenothiazine and terminal benzothiazole *θ*_3_ was 15.02° for the major component in form I and 197.17° in form II (Table S6). In the most stable structure of a single molecule in a vacuum obtained by theoretical calculation, *θ*_3_ was 35.28°, indicating that the conformations of forms I and II slightly deviated from the most stable structure. The calculated total energies of 1 in each crystal structure and the energies of a unit cell with periodic boundary conditions were almost identical for both forms (Table S7). This result implied difficulty in discussing the thermodynamic stability of the crystal structures on the basis of theoretical calculations.

### SC–SC structural transition from achiral to chiral crystal structures

Differential scanning calorimetry (DSC) measurements of form I revealed an endothermic peak corresponding to the melting point at 210 °C (Fig. 3a). In contrast, form II exhibited a small endothermic peak of 3.09 kJ mol^−1^ at 159 °C in addition to the melting point. This small endothermic peak was observed solely during the 1st heating, and only the melting point at 210 °C was observed during the 2nd heating. Variable temperature PXRD (VT-PXRD) measurements of form II revealed that the small endothermic peak was attributed to the structural transition from form II to I. The diffraction peaks corresponding to form I began to appear at approximately 150 °C, whereas the intensity of the peaks corresponding to form II decreased (Fig. 3b). At 230 °C, the peaks completely disappeared due to melting. The structural transition from form II to I was not observed upon repeated heating and cooling. Variable temperature microscopy observations using a single crystal of form II clearly revealed the structural transition. From the DSC and VT-PXRD measurements, the structural transition point was estimated to be 150–160 °C. However, the single crystals did not necessarily undergo the transition at 150–160 °C and sometimes maintained a superheated state.

Fig. 3c shows polarized optical microscopy (POM) images of a sample that underwent a transition at approximately 180 °C. In most cases, once the structural transition begins from a certain point, the transition propagates throughout the entire crystal. Under POM, this change was clearly observed as a change in the interference colour. While the propagation speed varied depending on the crystal size, in most cases, it was completed within a few seconds to tens of seconds. This moderate propagation speed allowed the maintenance of single crystallinity without any salient effects. Single crystallinity was confirmed by SCXRD after the transition. Although the morphology of the crystal after the transition was different from that of the crystal prepared from solution, the crystal structure was identical to that of form I. The orientation of the crystal before and after the structural transition was determined by the face index, revealing that the broad face of the plate crystal was corresponded to the (001) plane of the racemic compound and that it turned (001) plane of the conglomerate after the structural transition. Under fluorescence microscopy, the transition was observed as the colour changed, as shown in Fig. S1. Green fluorescence was observed at the edge of the crystals before the transition, and the colour slightly turned light green throughout the transition.

When a form II crystal was heated with solid-seeding of a form I crystal, the structural transition progressed from the point of contact, resulting in a crystal with the same chirality as the seed (Fig. 3e–g). Notably, the structural transition did not always initiate from the contact point; sometimes, the transition began from other points (Fig. 3h), and it rarely simultaneously occurred from multiple points (Fig. 3i). Focusing on cases in which the transitions occurred from contact points, the chirality of the crystal after the transition was the same as that of the seed crystal. This chirality transfer from a seed crystal was conducted using an *R* or *S* crystal 10 times each, and the chirality after the transition was successfully controlled with over 95% probability (Table S3 and SI movies). When the transition initiated from other points rather than contact points, the chirality after the transition was random.

### Optical properties

**Fig. 4:**
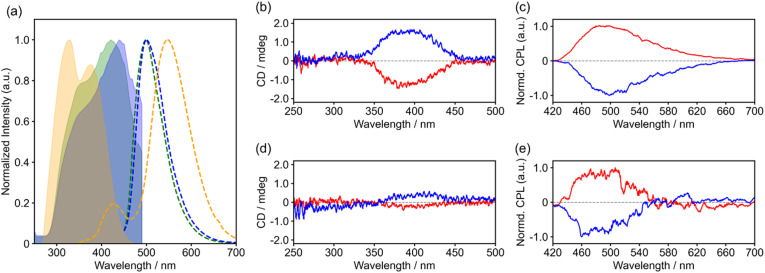
(a) Emission and excitation spectra, displayed as dotted lines and shaded areas, respectively. Yellow: CHCl_3_ solution (excited at 330 nm for the emission spectrum, observed at 550 nm for the excitation spectrum); blue: form I (excited at 385 nm, observed at 500 nm); green: form II (excited at 385 nm, observed at 500 nm). (b) CD and (c) CPL (excited at 350 nm) spectra for form I (red: *R*-isomer and blue: *S*-isomer). (d) CD and (e) CPL spectra (excited at 350 nm) for the crystals after the structural transition (red: *R*-isomer and blue: *S*-isomer).

The fluorescence and excitation spectra of 1 in the chloroform solution revealed emission with a maximum wavelength of 550 nm, which was attributed to intramolecular charge transfer (CT) (Fig. 4a). Both forms I and II in the solid-state exhibited fluorescence spectra with a maximum wavelength of 500 nm, and the form I spectrum had a shoulder band on the longer wavelength side. The fluorescence quantum yield *Φ*_FL_ was 0.27 for both forms. Excitation spectrum measurements revealed clear differences in the maximum wavelengths, with peaks observed at 440 nm for form I and 420 nm for form II. The difference between the solution and solids can be attributed to the conformations that change the energy gaps corresponding to the intramolecular CT. This difference was also supported by the time-dependent density functional theory (TD-DFT) calculations based on the crystal structures (Fig. S3 and 4).

Single-crystal samples with predetermined chirality were diluted with KBr, and diffuse transmission circular dichroism (CD) measurements were performed. The spectral profiles of the enantiomeric pairs of the samples were mirror images (Fig. 4b). To avoid confusion with false signals arising from the linear dichroism (LD) component of the crystal, the LD was simultaneously measured, confirming that the effects of sample anisotropy were negligible (Fig. S8). The CD spectrum showed a maximum at approximately 390 nm, with a symmetric profile corresponding to the crystal chirality, which appeared at the same position as the peak in the solid-state excitation spectrum.

The CPL spectrum was also measured using a crystalline sample diluted with KBr. The CPL bands peaked at approximately 500 nm, which was comparable to the position of the corresponding PL band (Fig. 4c). The average *g*_lum_ value at approximately 500 nm was 8.9 × 10^−4^. Although not the highest reported value, it is moderate compared with those of other reported organic CPL materials. Similarly, small organic molecules such as single [4]helicenes and [5]helicene fragments are in the order of 10^−3^.^48^

Furthermore, these chiroptical properties emerged from structural transitions (Fig. 4d and e) as the materials were crystallized into chiral crystals. The original compound was a racemic crystal; thus, it did not exhibit CD and CPL before the structural transition. Chiroptical properties emerged as the entire crystal transformed into a single chirality by the structural transition.

## Discussion

A detailed comparison of the crystal structures before and after the structural transition revealed that the SC–SC transition involved conformational changes and drastic molecular rearrangements. Although the exact sequence of movements remains unclear, the conformational changes included (1) C–C bond rotation between the benzothiazole and phenylene groups and (2) chirality inversion associated with the phenothiazine ring. These conformational changes proceeded continuously or simultaneously. The molecular orientation was turned by approximately 90° based on the face index by SCXRD, which means that the alignment of the molecular short and long axes was reversed.

The C–C bond rotation between the benzothiazole and phenylene groups was suggested by the inversion of the *syn*/*anti* ratio. The benzothiazole ring was analysed as the disordered structures, *syn*- and *anti*-forms, which were generated by approximately 180° rotation of the C–C bond. When the occupancy ratios before and after the structural transition were compared, the *syn*/*anti* ratio was inverted (Table S4). This result suggests the 180° rotation of the C–C bond during the structural transition. The rotational barrier of the C–C bond was calculated to be 19.7 kJ mol^−1^, which allows free rotation in solution or in a vacuum. Although the same criteria cannot be applied in the crystalline state, the thermal vibration of the molecules is sufficiently large at 150 °C; thus, the rotation sufficiently occurs in the space created by the rearrangement of the molecules.

Regarding the chirality inversion during the transition, the inversion barrier of the phenothiazine ring was calculated using a model compound, 3,10-dimethyl-10*H*-phenothiazine, to be 27.7 kJ mol^−1^. This value was sufficient for free inversion in the crystal if there was enough space (Fig. S2 and Table S8). The transition state for chirality inversion, shown in Fig. S6, demonstrates a non-chiral intermediate with the phenothiazine ring adopting a nearly planar conformation.

**Fig. 5:**
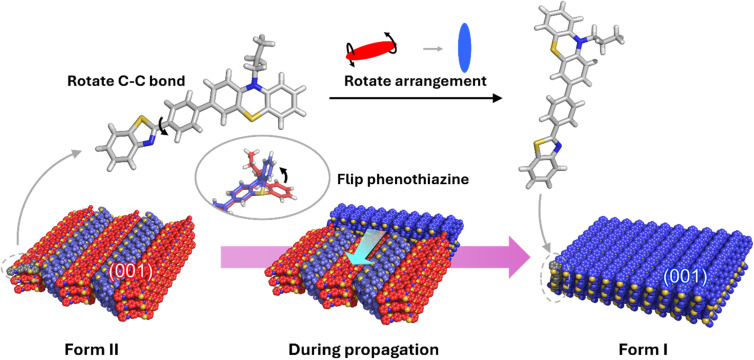
Schematic illustration of the structural transition. During the transition, racemization by the flipping of phenothiazine, rotation of benzothiazole, and drastic changes in the molecular arrangement occurred.

Compared with these conformational changes, the rearrangement of the molecules was surprisingly drastic. Fig. 5 shows a schematic illustration of the structural transition. The (001) plane of the racemic compound became the (001) plane of the conglomerate after the transition. Since the space group of the racemic compound is *Pna*2_1_, the (001) plane is perpendicular to the 2_1_-axis. In contrast, the space group of the conglomerate is *P*2_1_; thus, the 2_1_-axis was not in the original [001] direction. Before the transition, the long molecular axis was arranged in the in-plane direction, but after the transition, the long molecular axis was oriented perpendicular to the plane, indicating that the molecules had rotated nearly 90°. This result is consistent with the differences in interference colours observed in POM images before and after the structural transition (Fig. 3c). The long molecular axis was aligned perpendicular to the transmitted light before the transition, resulting in a high degree of anisotropy relative to the polarized light, and strong interference colours were observed depending on the slight differences in the crystal thickness. In contrast, after the transition, the long molecular axis was aligned parallel to the transmitted light, resulting in low anisotropy, and the interference colours appeared nearly colourless. Generally, structural transitions involving such large packing changes often result in the loss of single crystallinity due to the nucleation and growth processes occurring in the crystal. Therefore, the retention of single crystallinity during such a structural transition is extremely rare.^49,50^

On the basis of the structural changes observed above, the mechanism of the structural transition is considered as follows. At the transition temperature, the thermal vibration of the molecules and the crystalline lattice generate transient spaces around the molecules, which enables the drastic rearrangement of the molecules and the inversion of the phenothiazine. The C–C bond rotation simultaneously proceeded with the rearrangement. Then, molecules adjacent to the nucleus are incorporated into the crystal and the transition propagates. When solid-seeding was applied and the transition propagated from the contact point, it is considered that the surrounding molecules were incorporated in accordance with the chirality of the seed crystal. In contrast, when the transition initiated from a site other than the contact point, transient spaces formed around the molecules enabled random inversion of the phenothiazine, resulting in a chirality fluctuation state. In such a state, it is presumed that a local domain consisting of molecules with the same chirality was spontaneously formed, which then served as a nucleus for the transition. From the viewpoint of nonequilibrium thermodynamics, this phenomenon involves the dissipation of heat introduced into the system through molecular vibrations and racemization, and the convergence towards one chirality by the structural transition proceeded at some point. That is, spontaneous CSB was observed upon the SC–SC structural transition.

The CPL spectra of the crystals after the structural transition indicated that the racemic compound transformed into a conglomerate with a single enantiomer, supporting the occurrence of CSB in a single crystal. The value of *g*_lum_ for form I was 8.9 × 10^−4^ (at 500 nm), which is near to that of many helicene derivatives.^48^ This excellent CPL property can be triggered by the structural transition; therefore, 1 could be applied as a thermally stimulus-responsive single crystalline CPL material.

To our knowledge, this is the first example of complete spontaneous CSB upon SC–SC transformation of molecular crystals. Even in terms of molecular-level CSB, the present phenomenon is only the third example after Viedma ripening and preferential enrichment. In addition, the convergence of molecular chirality over an entire crystal through chirality inversion during SC–SC transformation has not been reported thus far. Notably, the present phenomenon occurred in the simplest system compared with the previous two examples: Viedma ripening and preferential enrichment. The previous examples occurred in solid–liquid systems, *i.e.*, mixtures of two or more components on different scales. The present phenomenon completely occurred within a single crystal, *i.e.*, the system was composed of a single component. This feature makes the system an appropriate model system for theoretical research on CSB, which could lead to a detailed understanding of its mechanism.

## Conclusions

We discovered molecular-level spontaneous CSB through SC–SC transformation. The metastable racemic compound of chiral phenothiazine underwent a structural transition to a conglomerate upon heating in an SC–SC manner. This SC–SC transition was characterized by SCXRD measurement, POM observation, CD and CPL spectroscopy. Crystallographic analysis revealed that chirality inversion occurred in a single enantiomer, with convergence to single chirality. This complete CSB in the solid-state resulted in an excellent turn-on type CPL crystalline material. Furthermore, we demonstrated that chirality transfer from a crystal with a predetermined chirality could be used to control the chirality of the structural transition. Our findings provide the simplest model of molecular-level CSB that is self-contained within a system composed of a single component without any solvent molecules. In addition to its application as a CPL material, this material could serve as an experimental model for verifying theoretical studies on CSB.

## Experimental

### Preparation of forms I and II

Form I was prepared by slow evaporation from a mixture of chloroform and heptane (1/1, v/v). Form II was prepared by slow evaporation from ethyl acetate.

### Chirality transfer upon the structural transition from form I

A single crystal of dried form II was placed on a glass slide. A single crystal of form I whose chirality was predetermined by SCXRD was placed in contact with the form II crystal. The glass slide was placed on a temperature-controlled stage (YONEKURA MFG. Co., Ltd, MHO-300-2). The sample was monitored by POM (Leica Microsystems, DM4). The temperature of the stage was heated to 150 °C at 5 K min^−1^, and then the heating rate was decreased to 1 K min^−1^ until a structural transition occurred.

### CPL spectroscopy

A single crystal prepared from CH_2_Cl_2_/heptane solution, or a crystal after structural transformation, was gently ground and pressed with KBr to prepare a pellet using a ClearDisk CD-05 (JASCO). To suppress the influence of artifacts, the KBr pellet was carefully adjusted to be as thin as possible (*ca.* 10 μm). CPL spectra were recorded using a CPL spectrometer (JASCO CPL-300). The spectra were recorded a few times on the different regions such as the front and back sides of the sample to avoid any anisotropy effects or artifacts on signal interpretation.

## Author contributions

R. O. conceived the idea, performed the screening of candidate molecules, performed SCXRD, performed CD spectroscopy, created the figures, supervised the investigation, and wrote manuscript. M. O. prepared samples and performed SCXRD, PXRD, microscopic observations, chirality transfer experiments, and wrote manuscript. K. T., H. S., T. K. and T. N. discussed, performed and analyzed CPL spectroscopy. I. H. supervised the investigation and wrote manuscript. All authors discussed the results, and edited the final manuscript.

## Conflicts of interest

There are no conflicts to declare.

## Supplementary Material

SC-016-D5SC02623G-s001

SC-016-D5SC02623G-s002

SC-016-D5SC02623G-s003

SC-016-D5SC02623G-s004

SC-016-D5SC02623G-s005

SC-016-D5SC02623G-s006

SC-016-D5SC02623G-s007

SC-016-D5SC02623G-s008

SC-016-D5SC02623G-s009

SC-016-D5SC02623G-s010

SC-016-D5SC02623G-s011

SC-016-D5SC02623G-s012

SC-016-D5SC02623G-s013

SC-016-D5SC02623G-s014

SC-016-D5SC02623G-s015

SC-016-D5SC02623G-s016

SC-016-D5SC02623G-s017

SC-016-D5SC02623G-s018

SC-016-D5SC02623G-s019

SC-016-D5SC02623G-s020

SC-016-D5SC02623G-s021

SC-016-D5SC02623G-s022

SC-016-D5SC02623G-s023

SC-016-D5SC02623G-s024

## Data Availability

Source data for PXRD, DSC, VTPXRD, UV-vis spectra, excitation spectra, and chiroptical spectra are available at figshare at https://doi.org/10.6084/m9.figshare.28746668. Crystallographic data for forms I and II have been deposited at the CCDC under 2402055 and 2402056. CCDC 2402055 and 2402056 contain the supplementary crystallographic data for this paper.^51,52^ The data supporting this article have been included as part of the SI: general remarks, synthesis and preparation of crystals, crystallography, chirality transfer experiments, and theoretical calculations. See DOI: https://doi.org/10.1039/d5sc02623g.
