# Supplementary material for: Spontaneous chiral symmetry breaking in a single crystal
Source: Chem Sci. 2025 Aug 19;16(38):17621–9. doi: 10.1039/d5sc02623g (PMC12362298; doi:10.1039/d5sc02623g)
Supplement: SC-016-D5SC02623G-s012 [file SC-016-D5SC02623G-s012.pdf]

Supplementary Information (SI) for Chemical Science.
This journal is © The Royal Society of Chemistry 2025

Spontaneous chiral symmetry breaking in a single crystal

Table of Contents

General remarks (page 2)

Synthesis and preparation of crystals (page 3)

Crystallography (page 6)

Chirality transfer upon the structural transition from form I (page 9)

Fluorescence microscope images during the structural transformations (page 9)

Occupancy of the disordered structure before and after the structural transformations (page 10)

Theoretical calculation (page 12)

CPL properties and LD spectra (page 17)

Atomic coordinate for calculations (page 18)

Reference (page 37)

General remarks

All reagents and solvents were used as received from commercial suppliers. ^{1}H NMR spectra were recorded on a Bruker AV400M (400 MHz) spectrometer. The chemical shift of Tetramethylsilane (TMS) or chloroform was used as internal standards for the measurement: $\delta = 0.00$ or 7.26. Powder X-ray diffraction pattern was collected with Rigaku MiniFlex600. Differential scanning calorimetry (DSC) measurement was conducted with Rigaku Thermo plus EVO2 DSCvesta. Absorption spectra was recorded with JASCO V-770 spectrophotometer. Photoluminescence (PL) and excitation spectra were measured with JASCO FP-6500 spectrofluorometer. Diffuse transmission CD spectra were measured using JASCO J-1500 spectrometer. For CPL spectroscopy, a single crystal prepared from CH_2Cl_2/heptane solution, or a crystal after structural transformation was gently ground and pressed with a KBr. CPL spectra were recorded using JASCO CPL-300 spectrometer. The spectra were recorded a few times on the different regions of the sample to avoid any anisotropy effects on signals interpretation.

Synthesis

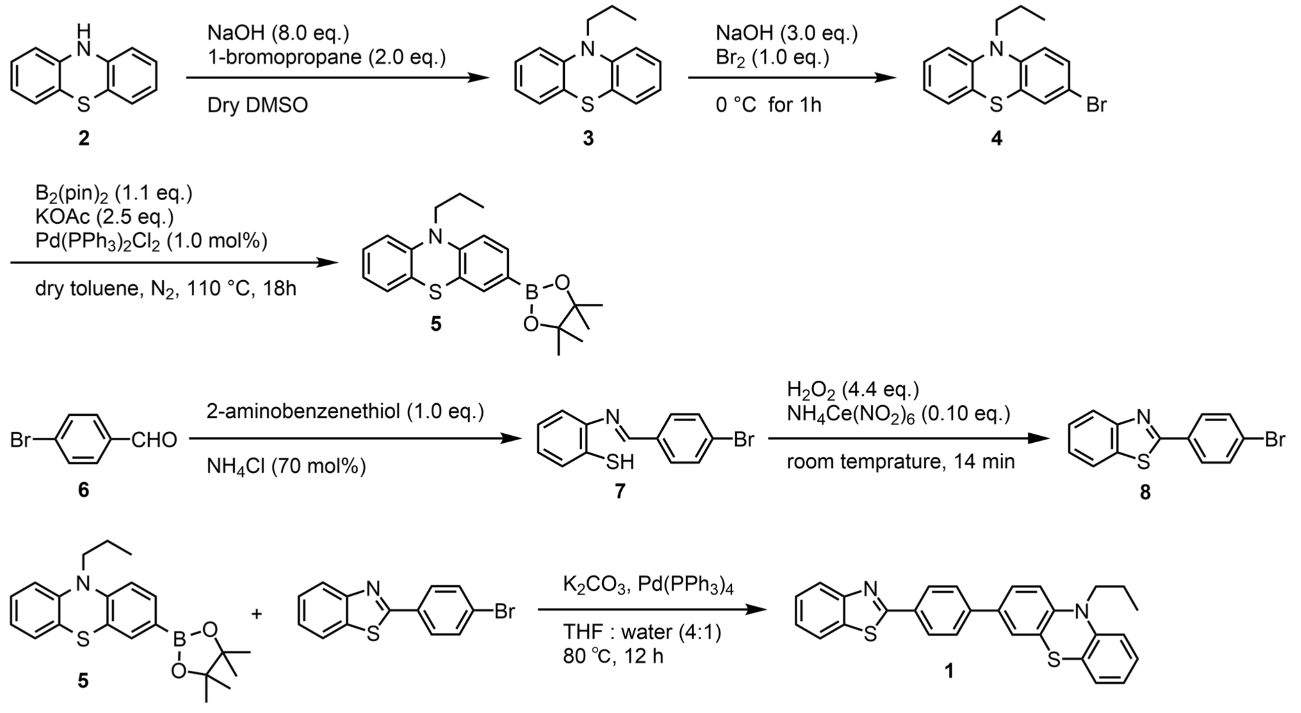

Scheme S1. Synthesis of phenothiazine derivative **1**.

N-Propylphenothiazine (3)

To a 200 mL three-neck flask, 2 (4.99 g, 25.0 mmol) and dimethyl sulfoxide (35 mL) were added. A solution of sodium hydroxide (7.97 g, 0.199 mol) in dimethyl sulfoxide (40 mL) was added to the reaction mixture and stirred under a nitrogen atmosphere at 90 °C for 16 hours. After cooling to room temperature, the undissolved sodium hydroxide was removed by suction filtration. The reaction mixture was poured over ice, followed by the addition of water (500 mL). The solution was extracted three times with ethyl acetate (150 mL). The combined organic layers were washed with saturated brine, dried over $MgSO_4$, and concentrated under reduced pressure to obtain a brown oil (7.07 g). The product was purified by silica gel chromatography (hexane) to afford the compound **3** as a white solid (5.35 g, 89%). ^{1}H NMR (400 MHz, $CDCl_3$, 30 °C): δ 7.13 (m, 4H), 6.89 (t, *J* = 7.6, 7.4 Hz, 2H), 6.85 (d, *J* = 8.0 Hz, 2H), 3.81 (t, *J* = 7.2 Hz, 2H), 1.80 (m, 2H), 1.00 (t, *J* = 7.4 Hz, 3H).

3-Bromo-10-propyl-10H-phenothiazine (4)

To a 500 mL three-neck flask, **3** (210 mg, 0.870 mmol) and chloroform (2.30 mL) were added. A solution of sodium hydroxide (0.491 g, 12.3 mmol) in acetic acid (11.9 mL) was added to the reaction mixture, then cooled to 0 °C. A mixture of bromine (43 μL, 0.829 mmol) and acetic acid (1.23 mL) was added dropwise at 0 °C, and the reaction was stirred for 1 hour. Saturated aqueous sodium bicarbonate was added until neutralized, and the solution was extracted twice with dichloromethane (50 mL). The combined organic layers were washed with saturated brine, dried over $MgSO_4$, and

concentrated under reduced pressure to obtain a yellow oil (0.257 g). The product was purified by silica gel chromatography (hexane) to afford the compound **4** as a yellow oil (0.178 g, 64%). ^{1}H NMR (400 MHz, $CDCl_3$, 30 °C): δ 7.34 (d, *J* = 7.6 Hz, 2H), 7.20 (m, 1H), 7.14 (d, *J* = 7.6 Hz, 1H), 7.01 (t, *J* = 6.4 Hz, 1H), 6.95 (m, 2H), 3.81 (t, *J* = 6.9 Hz, 2H), 1.67 (m, 2H), 0.921 (m, 3H).

10-Propyl-3-(4,4,5,5-tetramethyl-1,3,2-dioxaborolan-2-yl)-10*H*-phenothiazine (5)

To a 300 mL three-neck flask, **4** (2.43 g, 7.59 mmol), toluene (150 mL), bis(pinacolato)diboron (2.61 g, 11.4 mmol), and potassium acetate (1.14 g, 19.0 mmol) were added. The solution was degassed by three freeze-pump-thaw cycles under a nitrogen atmosphere. $Pd(PPh_3)_2Cl_2$ (0.325 g, 0.463 mmol) was added, and the reaction mixture was stirred at 110 °C for 20 hours. After cooling to room temperature, water (150 mL) was added, and the solution was filtered through Celite to remove catalyst residues. The aqueous phase was extracted four times with dichloromethane (150 mL). The combined organic layers were washed with saturated brine, dried over $MgSO_4$, and concentrated under reduced pressure to obtain a dark green oil (3.751 g). The product was purified by silica gel chromatography (hexane/chloroform = 1/1, v/v) to afford the compound **5** as a red oil (1.22 g, 3.32 mmol, 44%). ^{1}H NMR (400 MHz, $CDCl_3$, 30 °C): δ 7.55 (s, 1H), 7.37 (t, *J* = 7.7 Hz, 1H), 7.11 (m, 2H), 6.89 (t, *J* = 7.3 Hz, 1H), 6.83 (m, 2H), 3.82 (s, 2H), 1.82 (m, 2H), 1.32 (s,12H), 0.995 (t, *J* = 7.4 Hz, 3H).

2-[[(4-Bromophenyl)methylene]amino]benzenethiol (7)

To a 100 mL Erlenmeyer flask, **6** (1.03 g, 5.57 mmol), a mixed solvent (methanol/water = 15/1, v/v, 30 mL), 2-aminothiophenol (0.69 mL, 6.48 mmol), and ammonium chloride (0.206 g, 3.85 mmol) were added, and the mixture was stirred for 90 minutes. Water (30 mL) was added, and the precipitate was collected by suction filtration and dried under reduced pressure to obtain a white solid (1.06 g). The solid was recrystallized from methanol (27 mL) at 55 °C, and the crystals were collected by suction filtration and dried under reduced pressure to afford the compound **7** as a white solid (0.432 g, 1.48 mmol, 26%). ^{1}H NMR (400 MHz, $CDCl_3$, 30 °C): δ 7.49 (d, *J* = 8.5 Hz, 2H), 7.42 (d, *J* = 8.5 Hz, 2H), 7.04 (d, *J* = 7.5 Hz, 1H), 6.96 (td, *J* = 7.6, 1.20 Hz, 1H), 6.77 (td, *J* = 7.5, 0.96 Hz, 1H), 6.68 (d, *J* = 7.8 Hz, 1H), 6.35 (s, 1H).

2-(4-Bromophenyl)benzothiazole (8)

To a 100 mL Erlenmeyer flask, **7** (2.12 g, 7.25 mmol), acetonitrile (33 mL), hydrogen peroxide (4.32 mL, 43.2 mmol), and $NH_4Ce(NO_2)_6$ (0.602 g, 1.10 mmol) were added, and the mixture was stirred for 2 hours. Water (30 mL) was added, and the solution was extracted three times with ethyl acetate (30 mL). The combined organic layers were washed with saturated brine, dried over $MgSO_4$, and concentrated under reduced pressure to obtain a white solid (2.03 g). The solid was recrystallized from methanol (110 mL) at 55 °C, and the crystals were collected by suction filtration and dried under reduced pressure to afford the compound **8** as a brown solid (0.430 g, 1.48 mmol, 14%). ^{1}H NMR (400 MHz, $CDCl_3$, 30 °C): δ 8.07 (d, *J* = 8.3 Hz, 1H), 7.97 (d, *J* = 8.6 Hz, 2H), 7.91 (d, *J* = 8.0 Hz, 1H),

7.64 (d, J = 8.6 Hz, 2H), 7.51 (td, J = 8.6, 1.9 Hz, 1H), 7.41 (td, J = 7.5, 2.1 Hz, 1H).

3-(4-(benzo[d]thiazol-2-yl)phenyl)-10-propyl-10H-phenothiazine (1)

To a 100 mL three-neck flask, **5** (1.22 g, 3.32 mmol), a mixed solvent (THF/water = 4/1, v/v, 35 mL), **8** (0.963 g, 3.32 mmol), and potassium carbonate (2.31 g, 16.7 mmol) were added, and the solution was degassed by three freeze-pump-thaw cycles. $Pd(PPh_3)_4$ (0.372 g, 0.322 mmol) was added, and the reaction mixture was refluxed at 80 °C for 24 hours. After cooling to room temperature, water (40 mL) was added, and the solution was extracted three times with dichloromethane (50 mL). The combined organic layers were washed with saturated brine, dried over $MgSO_4$, and concentrated under reduced pressure to obtain a yellow solid (1.70 g). The product was purified by silica gel chromatography (hexane/dichloromethane = 7/3, v/v) to afford the compound **1** as a yellow solid (1.01 g, 2.24 mmol, 68%). ^{1}H NMR (400 MHz, $CDCl_3$, 30 °C): δ 8.13 (d, J = 8.1 Hz, 2H), 8.08 (d, J = 8.1 Hz, 1H), 7.92 (d, J = 7.6 Hz, 1H), 7.66 (d, J = 8.4 Hz, 2H), 7.50 (t, J = 8.0 Hz, 1H), 7.46–7.37 (m, 3H), 7.15 (d, J = 7.5 Hz, 2H), 6.95–6.87 (m, 3H), 3.86 (t, J = 7.0 Hz, 2H), 1.90–1.84 (m, 2H), 1.04 (t, J = 7.4 Hz, 3H).

Preparation of crystals

Form I was prepared by slow evaporation from a mixture of chloroform and heptane (1/1, v/v). Form II was prepared by slow evaporation from ethyl acetate.

Table S1. Crystallization conditions of **1** with various solvents.

Entry	Solvent	Deposited forms
1	CH_2Cl_2 : heptane = 1: 1	Form I and II
2	Chloroform : heptane = 1 : 1	Form I and II
3	CH_2Cl_2	Form I
4	Chloroform : heptane = 1 : 1	Form I
5	EtOAc	Form II
6	THF	Form I
7	Benezene	Form I
8	MTBE	Form I
9	Et_2O	Form I
10	Cyclohexane	Form I
11	MeOH	amorphous
12	EtOH	amorphous
13	iPrOH	amorphous

Crystallography

X-ray diffraction data were collected using a Rigaku XtaLAB P200 diffractometer, and the Mo-Kα line (λ = 0.71076 Å) was used as the X-ray source. The data collection was carried out with Rigaku CrystalClear software. The cell refinement and data reduction were carried out with CrysAlis PRO.[S1] The crystal structure were solved by intrinsic phasing method using SHELXT[S2] and refined on F^2 with all data using SHELXL-2014.[S3] All non-hydrogen atoms were refined anisotropically, and hydrogen atoms were placed in ideal positions and refined as rigid atoms with the relative isotropic displacement parameters. All calculations were carried out using Olex2 program.[S4] The programs Mercury were used for analysis and visualization.[S5] CCDC deposition number 2402055–2402056 contain the supplementary crystallographic data for this paper. These data are provided free of charge by The Cambridge Crystallographic Data Centre.

Table S2. Crystallographic data of the conglomerate and racemic compound.

	Form I (conglomerate)	Form II (racemic compound)
Chemical Formula	$C_{28}H_{22}N_2S_2$	$C_{28}H_{22}N_2S_2$
Formula mass	450.59	450.59
Crystal system	Monoclinic	Orthorhombic
Space group	$P2_1$	$Pna2_1$
a / Å	8.5534(3)	39.5906(7)
b / Å	5.5300(2)	7.17130(10)
c / Å	22.8598(7)	7.64230(10)
α / °	90	90
β / °	94.991(3)	90
γ / °	90	90
Unit cell volume / Å^3	1077.18(6)	2169.77(6)
Temperature / K	113	113
Z, Z'	2, 1	4, 1
No. of measured reflections	20477	40303
No. of independent reflections	5392	5315
R_{int}	0.0535	0.0405
Final R_1 values ($I > 2\sigma(I)$)	0.0688	0.0423
Final $wR(F^2)$ values (all data)	0.1881	0.0962
Goodness of fit on F^2	1.038	1.015
Flack parameter	0.07 (4)	0.00(2)
d / g·cm^{-3}	1.389	1.379
μ [Mo-Kα] / mm^{-1}	0.267	0.265
CCDC No.	2402056	2402055

Table S3. Crystallographic data of the sample after structural transformation. The absolute configuration of each single crystal was determined based on the Flack parameter.

	1	2	3	4	5	6	7	8	9	10
Chemical Formula	$C_{28}H_{22}N_2S_2$	$C_{28}H_{22}N_2S_2$	$C_{28}H_{22}N_2S_2$	$C_{28}H_{22}N_2S_2$	$C_{28}H_{22}N_2S_2$	$C_{28}H_{22}N_2S_2$	$C_{28}H_{22}N_2S_2$	$C_{28}H_{22}N_2S_2$	$C_{28}H_{22}N_2S_2$	$C_{28}H_{22}N_2S_2$
Formula mass	450.59	450.59	450.59	450.59	450.59	450.59	450.59	450.59	450.59	450.59
Crystal system	Monoclinic	Monoclinic	Monoclinic	Monoclinic	Monoclinic	Monoclinic	Monoclinic	Monoclinic	Monoclinic	Monoclinic
Space group	$P2_1$	$P2_1$	$P2_1$	$P2_1$	$P2_1$	$P2_1$	$P2_1$	$P2_1$	$P2_1$	$P2_1$
a / Å	8.5590(6)	8.5749(8)	8.5521(6)	8.5749(13)	8.5496(4)	8.5517(8)	8.5885(9)	8.5812(11)	8.587(3)	8.5802(5)
b / Å	5.5331(3)	5.5417(5)	5.5308(3)	5.5588(8)	5.5312(2)	5.5287(4)	5.5505(5)	5.5633(8)	5.5517(15)	5.5416(3)
c / Å	22.8637(14)	22.917(2)	22.8704(12)	22.947(3)	22.877(1)	22.8459(19)	22.934(2)	22.982(3)	22.968(6)	22.9169(10)
α / °	90	90	90	90	90	90	90	90	90	90
β / °	95.002(6)	95.087(9)	95.026(6)	95.186(14)	95.056(4)	95.002(8)	95.05(1)	95.017(12)	95.34(3)	95.132(4)
γ / °	90	90	90	90	90	90	90	90	90	90
Unit cell volume / $Å^3$	1078.65(12)	1084.72(17)	1077.61(11)	1089.3(3)	1077.63(8)	1076.04(16)	1089.03(18)	1093.0(3)	1090.2(6)	1085.29(10)
Temperature / K	113	113	113	113	113	113	113	173	113	113
Z	2	2	2	2	2	2	2	2	2	2
No. of measured reflections	11157	12218	14053	12207	12200	25557	13053	20813	11755	13375
No. of independent reflections	5090	5244	5236	5236	4888	5639	5238	5374	5177	5145
R_{int}	0.05	0.057	0.065	0.078	0.037	0.094	0.088	0.169	0.225	0.028
Final R_1 values ($I > 2\sigma(I)$)	0.063	0.071	0.07	0.103	0.054	0.087	0.12	0.153	0.114	0.042
Final $wR(F^2)$ values (all data)	0.162	0.192	0.184	0.304	0.141	0.217	0.314	0.396	0.299	0.113
Goodness of fit on F^2	1.02	1.03	1.04	1.09	1.07	1.05	1.17	1.06	0.87	1.04
d / $g \cdot cm^{-3}$	1.387	1.38	1.389	1.374	1.389	1.391	1.374	1.369	1.373	1.379
μ [Mo-Kα] / mm^{-1}	0.27	0.27	0.27	0.26	0.27	0.27	0.26	0.26	0.26	0.27
Flack parameter	0.09(7)	0.01(6)	0.00(9)	0.00(9)	0.03(4)	0.08(7)	0.04(9)	0.28(15)	0.1(3)	−0.02(3)
Chirality of the attached crystal	*S*	*S*	*S*	*S*	*S*	*S*	*S*	*R*	*S*	*S*
Chirality of the crystal	*S*	*S*	*S*	*S*	*S*	*S*	*S*	*R*	*S*	*S*

Table S3. Crystallographic data of the sample after structural transformation (continued).

	11	12	13	14	15	16	17	18	19	20	21
Chemical Formula	$C_{28}H_{22}N_2S_2$	$C_{28}H_{22}N_2S_2$	$C_{28}H_{22}N_2S_2$	$C_{28}H_{22}N_2S_2$	$C_{28}H_{22}N_2S_2$	$C_{28}H_{22}N_2S_2$	$C_{28}H_{22}N_2S_2$	$C_{28}H_{22}N_2S_2$	$C_{28}H_{22}N_2S_2$	$C_{28}H_{22}N_2S_2$	$C_{28}H_{22}N_2S_2$
Formula mass	450.59	450.59	450.59	450.59	450.59	450.59	450.59	450.59	450.59	450.59	450.59
Crystal system	Monoclinic	Monoclinic	Monoclinic	Monoclinic	Monoclinic	Monoclinic	Monoclinic	Monoclinic	Monoclinic	Monoclinic	Monoclinic
Space group	$P2_1$	$P2_1$	$P2_1$	$P2_1$	$P2_1$	$P2_1$	$P2_1$	$P2_1$	$P2_1$	$P2_1$	$P2_1$
a / Å	8.5861(8)	8.6164(12)	8.5974(8)	8.5906(12)	8.5557(4)	8.5782(14)	8.5504(5)	8.5582(4)	8.5564(7)	8.5537(3)	8.5575(4)
b / Å	5.5468(5)	5.5611(7)	5.5586(5)	5.5382(7)	5.5385(2)	5.5409(7)	5.5237(3)	5.5319(3)	5.5249(4)	5.5297(2)	5.5343(3)
c / Å	22.9172(14)	23.009(3)	22.973(2)	22.947(3)	22.9029(8)	22.935(3)	22.8554(9)	22.8667(9)	22.8663(16)	22.8677(8)	22.8936(11)
α / °	90	90	90	90	90	90	90	90	90	90	90
β / °	95.165(7)	94.777(15)	95.104(9)	95.090(12)	95.122(4)	95.105(13)	95.080(5)	95.052(4)	95.129(7)	95.034(4)	95.134(5)
γ / °	90	90	90	90	90	90	90	90	90	90	90
Unit cell volume / $Å^3$	1087.01(16)	1098.7(3)	1093.52(17)	1087.4(3)	1080.94(7)	1085.8(3)	1075.22(10)	1078.38(9)	1076.64(14)	1077.46(7)	1079.89(9)
Temperature / K	113	173	200	113	113	113	113	113	113	113	113
Z	2	2	2	2	2	2	2	2	2	2	2
No. of measured reflections	12542	21536	14232	18385	25841	14348	11212	14409	20207	11477	21039
No. of independent reflections	5087	5546	4849	5142	5660	5155	4975	5281	4943	4928	5196
R_{int}	0.076	0.129	0.059	0.095	0.045	0.114	0.046	0.033	0.07	0.039	0.065
Final R_1 values ($I > 2\sigma(I)$)	0.113	0.181	0.066	0.061	0.045	0.093	0.066	0.043	0.059	0.05	0.087
Final $wR(F^2)$ values (all data)	0.305	0.49	0.175	0.12	0.115	0.22	0.176	0.127	0.143	0.134	0.243
Goodness of fit on F^2	1.08	1.76	1.03	1.01	1.03	1.03	1.05	0.57	1.04	1.03	1.12
d / $g \cdot cm^{-3}$	1.377	1.362	1.368	1.376	1.384	1.378	1.392	1.388	1.39	1.389	1.386
μ [Mo-Kα] / mm^{-1}	0.27	0.26	0.26	0.27	0.27	0.27	0.27	0.27	0.27	0.27	0.27
Flack parameter	−0.08(10)	0.11(12)	0.06(8)	0.06(8)	0.01(3)	0.04(14)	0.20(5)	−0.01(4)	0.03(6)	0.22(5)	0.02(5)
Chirality of the attached crystal	*S*	*S*	*R*	*R*	*R*	*R*	*S*	*R*	*R*	*R*	*R*
Chirality of the crystal	*S*	*S*	*R*	*R*	*R*	*R*	*S*	*R*	*R*	*R*	*S*

Chirality transfer upon the structural transition from form I

A single crystal of dried form II was placed on a glass slide. A single crystal of form I whose chirality was predetermined by SCXRD was placed in contact with the form II crystal. The glass slide was placed on a temperature-controlled stage (YONEKURA MFG. Co., Ltd., MHO-300-2). The sample was monitored by POM (Leica Microsystems, DM4). The temperature of the stage was heated to 150 °C at 5 K min^{-1}, and then the heating rate was decreased to 1 K min^{-1} until a structural transition occured.

Fluorescence microscope images during the structural transformations

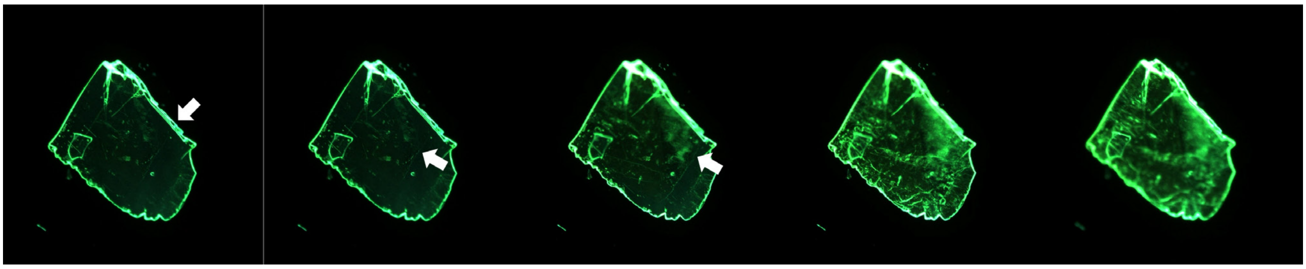

Figure S1. Fluorescence microscopy images under UV irradiation (λ_{ex} = 375 nm) at 180 °C.

Occupancy of the disordered structure before and after the structural transformations

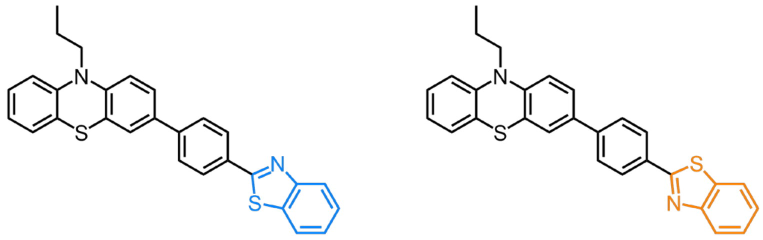

syn　　　　*anti*

Table S4. Occupancy ratio of *syn* and *anti*-conformers of **1** before and after the structural transformations.

Sample No.	*syn* : *anti* before the transition	*syn* : *anti* after the transition
21	0.19 : 0.81	0.82 : 0.18
22	0.19 : 0.81	0.82 : 0.18
23	0.20 : 0.80	0.88 : 0.12
24	0.19 : 0.81	0.84 : 0.16

Table S5. Crystallographic data for sample 21 to 24.

	21 before	21after	22 before	22 after	23 before	23 after	24 before	24 after
Chemical Formula	$C_{28}H_{22}N_2S_2$	$C_{28}H_{22}N_2S_2$	$C_{28}H_{22}N_2S_2$	$C_{28}H_{22}N_2S_2$	$C_{28}H_{22}N_2S_2$	$C_{28}H_{22}N_2S_2$	$C_{28}H_{22}N_2S_2$	$C_{28}H_{22}N_2S_2$
Formula mass	450.59	450.59	450.59	450.59	450.59	450.59	450.59	450.59
Crystal system	Orthorhombic	Monoclinic	Orthorhombic	Monoclinic	Orthorhombic	Monoclinic	Orthorhombic	Monoclinic
Space group	$Pna2_1$	$P2_1$	$Pna2_1$	$P2_1$	$Pna2_1$	$P2_1$	$Pna2_1$	$P2_1$
a / Å	39.6451(17)	8.5368(5)	39.6511(8)	8.5662(11)	39.6936(10)	8.5627(7)	39.7148(9)	8.5538(4)
b / Å	7.1810(3)	5.5222(3)	7.1785(2)	5.5325(8)	7.1713(2)	5.5394(5)	7.1503(2)	5.5289(2)
c / Å	7.6430(3)	22.8536(16)	7.6462(2)	22.874(3)	7.6467(2)	22.9184(16)	7.6382(2)	22.8389(8)
α, γ/ °	90	90	90	90	90	90	90	90
β / °	90	95.136(6)	90	95.087(12)	90	95.212(8)	90	95.115(4)
V / Å^3	2175.90(16)	1073.04(11)	2176.38(9)	1079.8(3)	2176.67(10)	1082.58(15)	2169.04(10)	1075.82(7)
Temp. / K	113	113	113	113	113	293	123	123
Z	4	2	4	2	4	2	4	2
No. of measured reflections	45576	25110	44488	26351	45560	12455	7906	6671
No. of independent reflections	6598	5489	6827	5513	6715	4845	3672	3270
R_{int}	0.123	0.087	0.083	0.205	0.099	0.071	0.033	0.037
Final R_1 values ($I > 2\sigma(I)$)	0.072	0.11	0.051	0.123	0.05	0.191	0.053	0.043
Final $wR(F^2)$ values (all data)	0.171	0.3	0.124	0.351	0.103	0.511	0.153	0.118
Goodness of fit on F^2	1.09	1.1	1	1.02	0.91	2.04	1.1	1.04
Flack parameter	−0.04(7)	0.08(7)	−0.03(4)	0.08(18)	0.00(5)	0.19(9)	0.02(3)	0.118(17)

Theoretical calculation

Optimization of hydrogen position in the experimentally obtained crystal structure of forms I and II were carried out with dispersion force corrected DFT method using periodic boundary condition on Quantum ESPRESSO 5.2.1 software.[S6–8] The Winmostar program was used as GUI.[S9] PBE functional with Grimme's dispersion correction method (D3 method) was used. The PSlibrary's ultrasoft pseudopotentials for PBE functional were used for all elements.[S10] The cutoff for wavefunctions (ecutwfc) and charge density (ecutrho) were gradually tightened, and the cutoff energy used in the final optimization were 64 and 576 Ry, respectively. The conversion threshold on total energy and forces for ionic minimization was used default value, 1.0D−4 and 1.0D−3, respectively. The conversion threshold for self-consistency calculation was 1.0D−8. K points were set at the gamma position except for the finial optimization, and 3×3×3 was used in the final optimization. Total energy of the final optimization was used for comparison of the energy for the crystal structures.

Optimized structures in the solid state were extracted from the optimized crystal structures described above. Optimized structures of *syn*- and *anti*-forms in vacuum condition were obtained by extracting a single molecule from the hydrogen-optimized crystal structure described above and performing full-atom structural optimization using Gaussian16 software.[S11] TD-DFT calculations were carried out at the CAM-B3LYP-D3/6-311+G** level of theory. The rotational barrier between the thiazole group and the phenylene group was determined by scanning the potential energy surface (PES) at the B3LYP-D3/6-311+G** level of theory. The inversion barrier of the phenothiazine ring was calculated using 3,10-dimethyl-10*H*-phenothiazine as a model by searching for the transition structure and performing IRC calculations, followed by determining the energy difference from the stable structure. The transition structure calculations and IRC calculations were conducted at the B3LYP/6-311+G** level of theory.

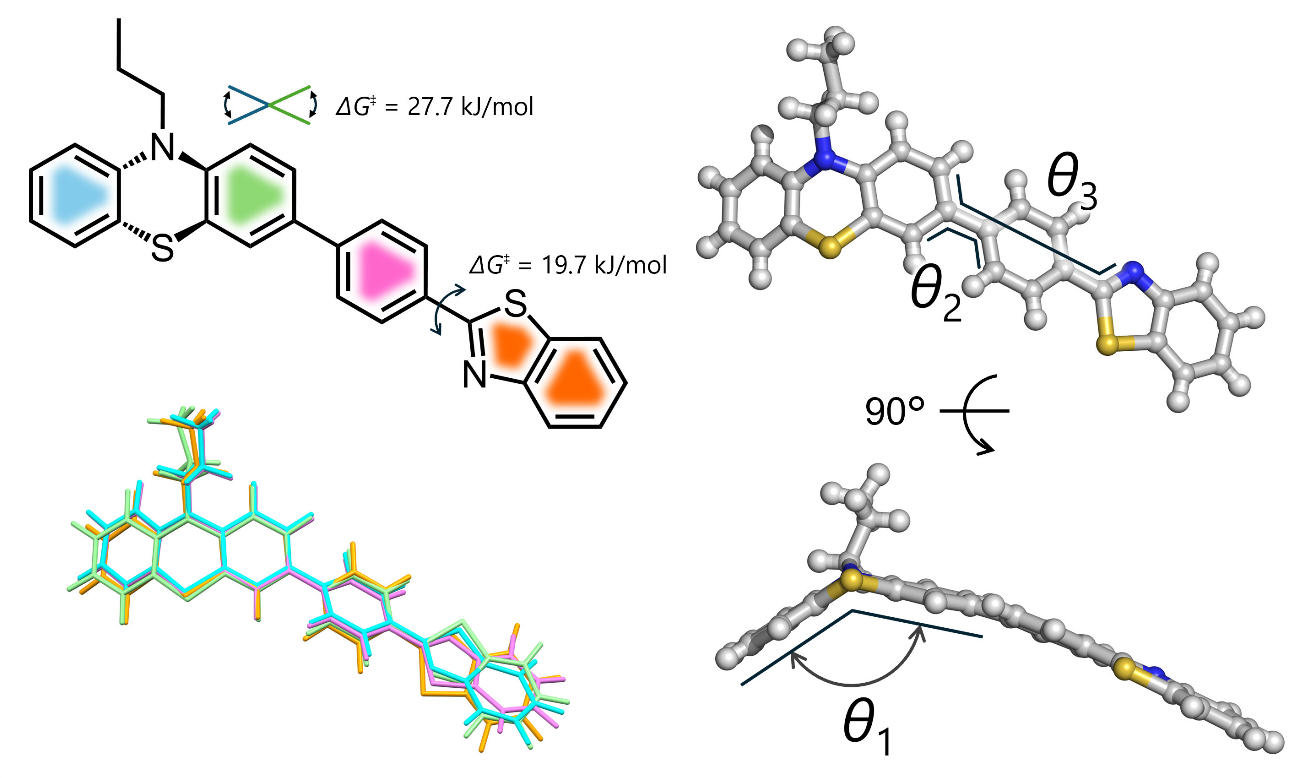

Figure S2. Comparison of conformers in crystals and optimized structures. θ_1–θ_3 indicate the bending angle of phenothiazine ring, dihedral angle between phenylene group and phenothiazine, dihedral angle between benzothiazole group and phenothiazine, respectively. The overlapped structure shows conformers in form I (orange), form II (green), optimized *syn*-conformer (pink), and optimized *anti*-conformer (cyan).

Table S6. The characteristic angles of the conformers.

	Form I / °	Form II / °	*syn* (optimized) / °	*anti* (optimized) / °
θ_1	135.57	149.58	137.20	137.20
θ_2	11.72	24.34	37.01	37.07
θ_3	15.02	197.17	35.28	217.10

Table S7. Total energies of the unit cell with periodic boundary conditions.

	Form I	Form II
Total energy for unit cell / Ry	−912.879	−1825.769
Energy per molecule /Ry	−456.439	−456.442

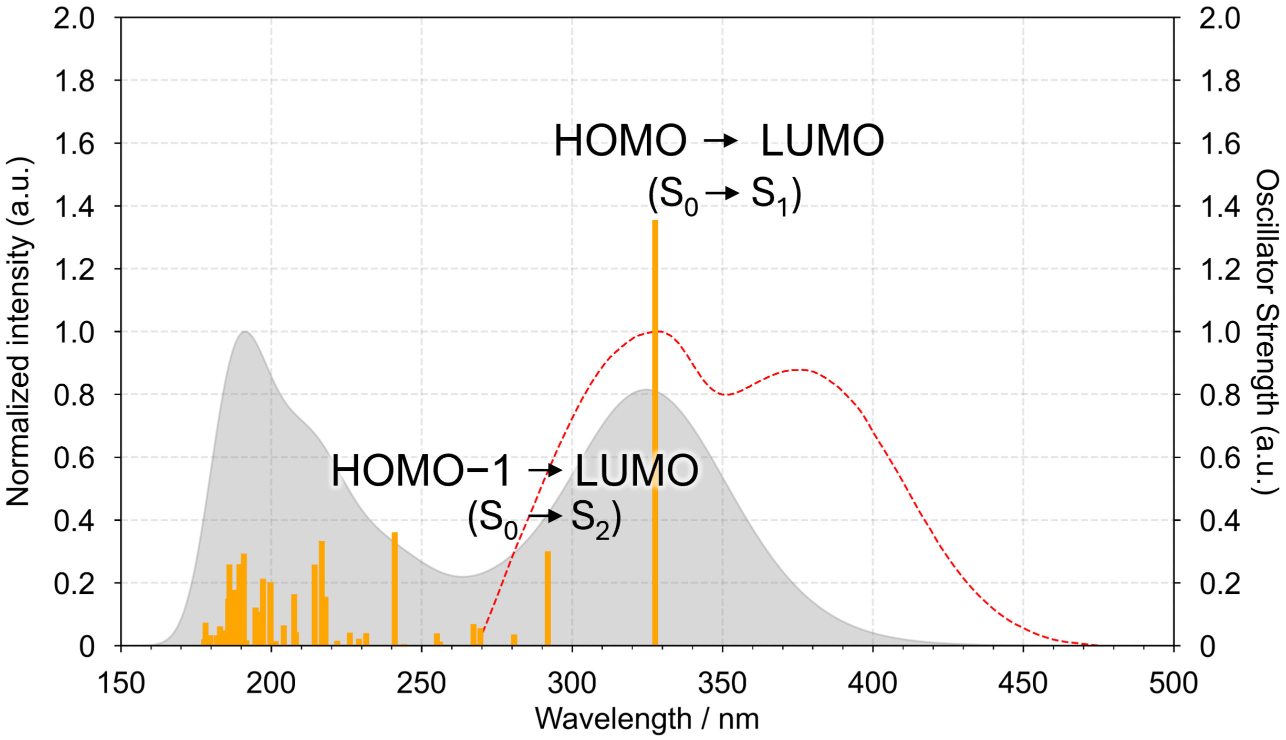

Figure S3. Experimental UV-Vis absorption spectrum of **1** in $CHCl_3$ (red dashed line), calculated absorption spectrum (gray shade), oscillator strength (orange bar).

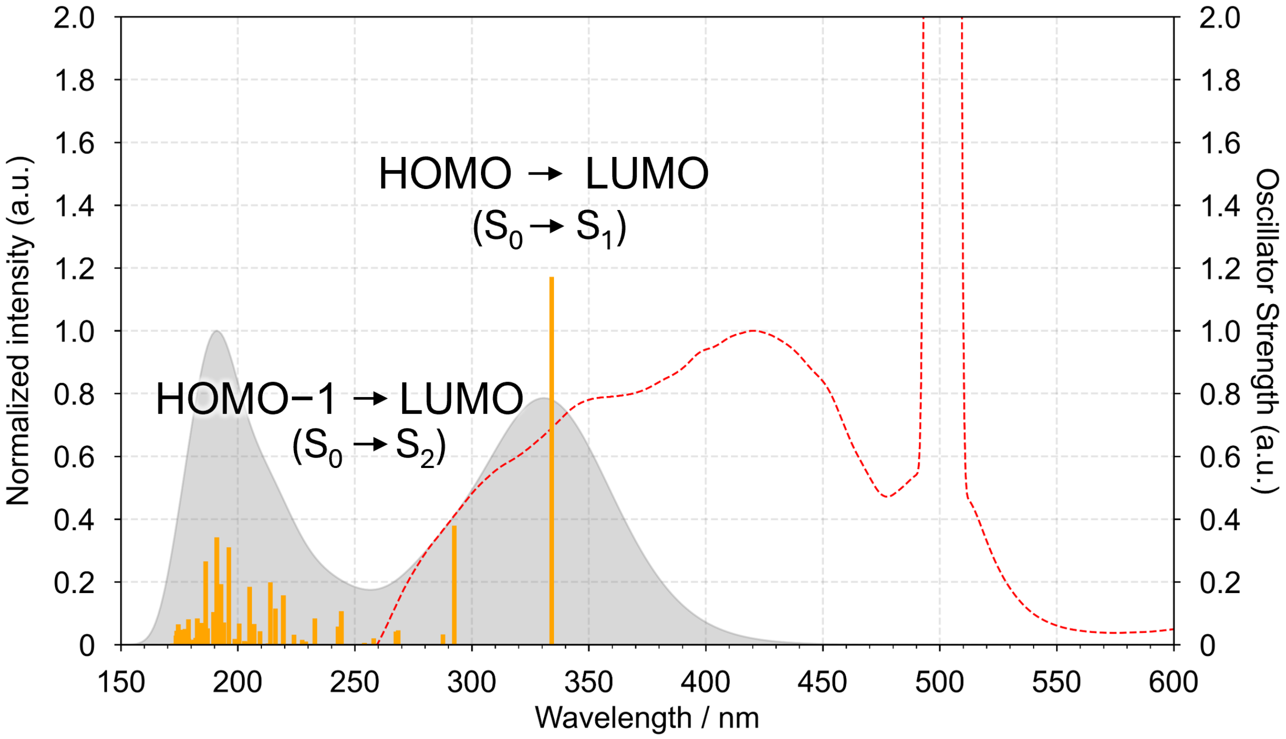

Figure S4. Experimental excitation spectrum of **1** in solid state (red dashed line), calculated absorption spectrum (gray shade), oscillator strength (orange bar).

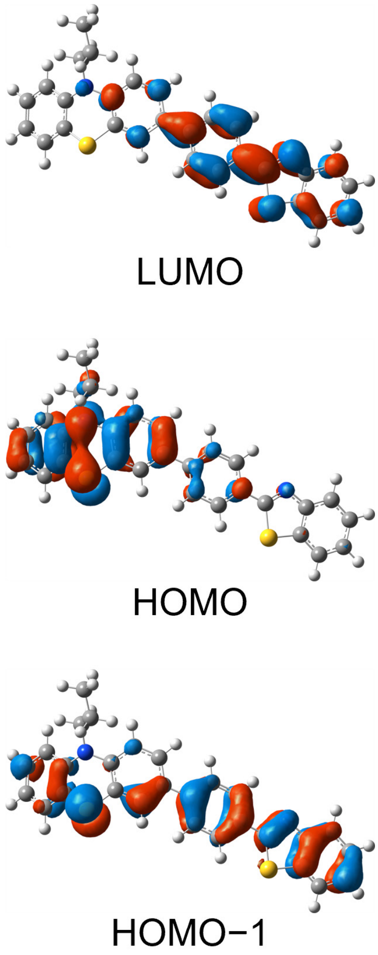

Figure S5. The calculated hole-electron distribution isosurfaces of **1**. (isovalue = 0.03)

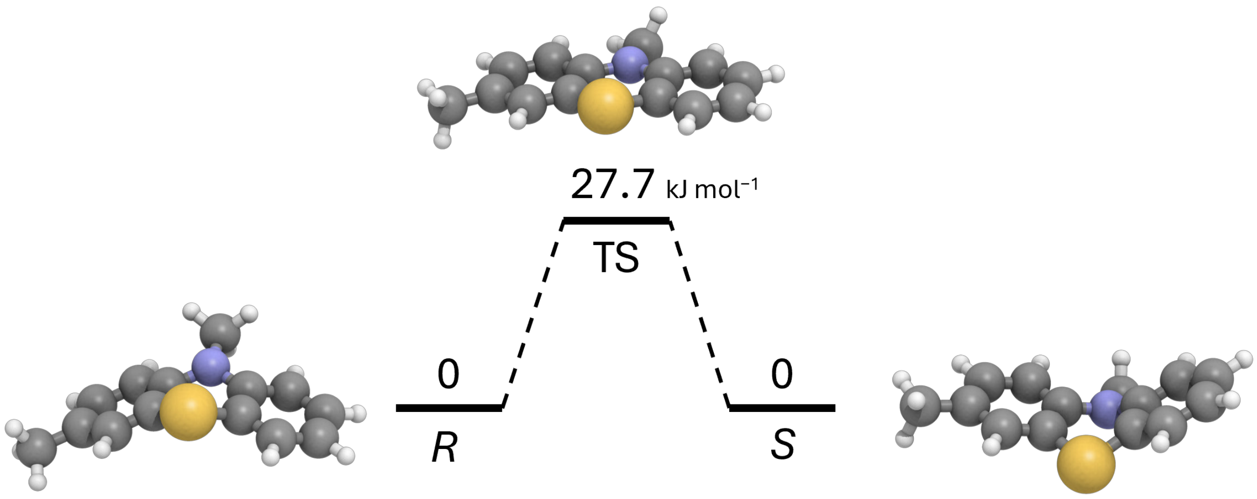

Figure S6. Energy diagram for the flipping of phenothiazine ring.

Table S8. Total energies of the stable state and transition state.

	R / *S*	TS
$E_{tot} + G_{corr}$/ kJ mol^{-1}	−2611459.79	−2611433.93
$\Delta G^{\ddagger}$ / kJ mol^{-1}	0	27.692

CPL properties and LD spectra

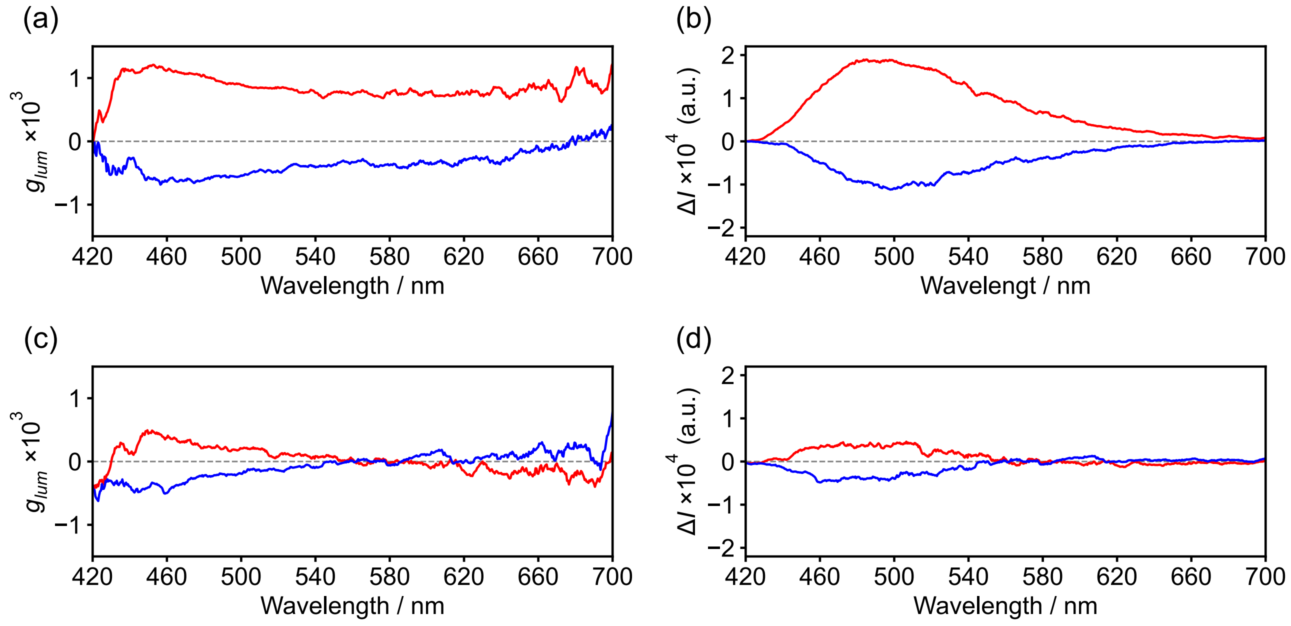

Figure S7. CPL properties for (a, b) Form I and (c, d) the sample after structure transition. (red: *R*-isomer, blue: *S*-isomer)

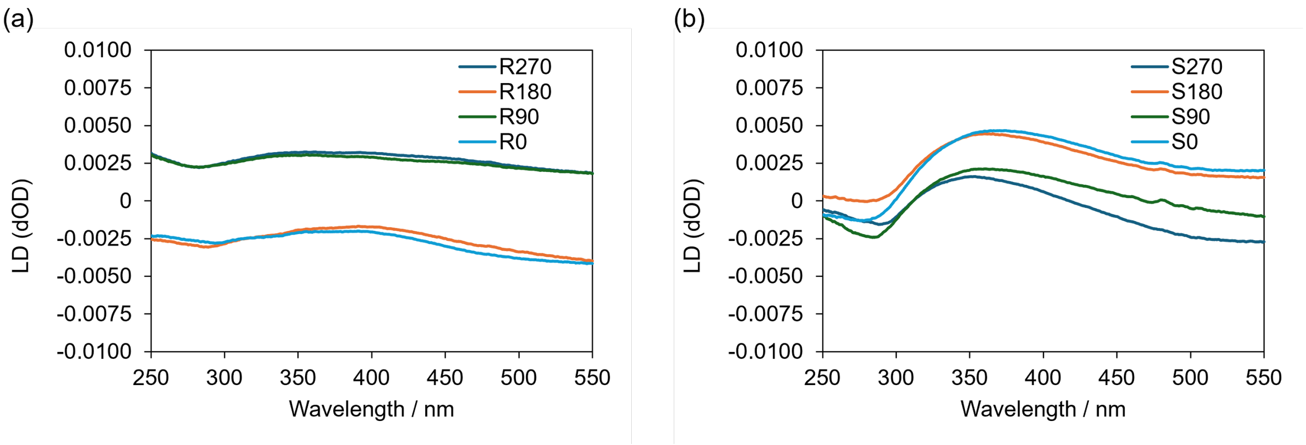

Figure S8. LD spectra of Form I for (a) *R*-isomer and (b) *S*-isomer with different sample holder angles. For all measurements, the amount of LD was below 0.005 that is enough low for CD spectra measurement.

Atomic coordinate for calculations

Fractional coordinate of form I

H	-0.388626318	0.159087618	-0.505926907
H	-0.314468531	0.752652731	-0.281280431
H	-0.492508035	0.131867119	-0.410207901
H	-0.183720903	0.847333976	-0.458281804
H	-0.290215098	0.825769892	-0.364011883
H	-0.598394128	0.136684833	-0.335627316
H	-0.865541000	0.529294780	-0.140714583
H	-0.865245244	0.345615993	-0.203433708
H	-0.137268677	0.773941650	-0.063966348
H	-0.668543685	0.085631246	-0.236698580
H	-0.098110390	0.495320909	-0.979094702
H	-0.280845711	0.149161846	-0.971518196
H	-0.709404212	0.220612658	-0.081886149
H	-0.699695193	0.037633400	-0.144180213
H	-0.016966489	0.221287052	-0.090652712
H	-0.999995242	0.013031573	-0.148632555
H	-0.114228122	0.276695410	-0.160168629
H	-0.497341792	0.082980071	-0.047090840
H	-0.881617912	0.848453481	-0.663803669
H	-0.867580961	0.585953082	-0.751264438
H	-0.025975894	0.211666583	-0.762573812
H	-0.201614006	0.089492772	-0.686415340
C	-0.399000000	0.474500000	-0.378270000
C	-0.367100000	0.304200000	-0.474320000
C	-0.394600000	0.607000000	-0.272980000
C	-0.426400000	0.291800000	-0.420190000
C	-0.251200000	0.688400000	-0.448130000
C	-0.275500000	0.502000000	-0.488900000
C	-0.312700000	0.675900000	-0.394130000
C	-0.551000000	0.264700000	-0.302800000
C	-0.450800000	0.450400000	-0.318100000
C	-0.340800000	0.588800000	-0.102380000
C	-0.535400000	0.392800000	-0.201220000
C	-0.437700000	0.580400000	-0.216210000
C	-0.862900000	0.340800000	-0.155530000
C	-0.216900000	0.623000000	-0.059740000

C	-0.592000000	0.234800000	-0.245950000
C	-0.195600000	0.466500000	-0.012140000
C	-0.296600000	0.274800000	-0.008070000
C	-0.445500000	0.395200000	-0.098380000
C	-0.708900000	0.226300000	-0.129590000
C	-0.005700000	0.205500000	-0.137740000
C	-0.421000000	0.237900000	-0.050870000
C	-0.205100000	0.515400000	-0.545210000
C	-0.136800000	0.396500000	-0.631200000
C	-0.949400000	0.683100000	-0.668700000
C	-0.046200000	0.609500000	-0.626040000
C	-0.943500000	0.536400000	-0.717580000
C	-0.033600000	0.322800000	-0.723800000
C	-0.131200000	0.252900000	-0.681710000
S	-0.372980000	0.793730000	-0.161560000
S	-0.076670000	0.749690000	-0.560250000
N	-0.571200000	0.368400000	-0.142410000
N	-0.225800000	0.348100000	-0.585060000
H	-0.611364724	0.659089485	-0.494071267
H	-0.685504071	0.252695925	-0.718727337
H	-0.507507496	0.631855436	-0.589794451
H	-0.816269845	0.347339317	-0.541718308
H	-0.709780533	0.325786562	-0.635982640
H	-0.401597349	0.636717636	-0.664365124
H	-0.134351111	0.029341190	-0.859262491
H	-0.134740958	0.845614550	-0.796565324
H	-0.862693116	0.273986152	-0.936032059
H	-0.331588869	0.585505426	-0.763329985
H	-0.901880471	0.995335666	-0.020908459
H	-0.719149928	0.649219486	-0.028493928
H	-0.290851430	0.721144477	-0.918127442
H	-0.299006877	0.537131504	-0.856012398
H	-0.983271973	0.720723800	-0.909373010
H	0.000216257	0.513166282	-0.851213073
H	-0.885785666	0.777250442	-0.839947124
H	-0.502770157	0.582819313	-0.952866398
H	-0.118373638	0.348466660	-0.336180920
H	-0.132421705	0.085953602	-0.248737199

H	-0.974010119	0.711705251	-0.237417874
H	-0.798350992	0.589532106	-0.313603313
C	-0.601000000	0.974500000	-0.621730000
C	-0.632900000	0.804200000	-0.525680000
C	-0.605400000	0.107000000	-0.727020000
C	-0.573600000	0.791800000	-0.579810000
C	-0.748800000	0.188400000	-0.551870000
C	-0.724500000	0.002000000	-0.511100000
C	-0.687300000	0.175900000	-0.605870000
C	-0.449000000	0.764700000	-0.697200000
C	-0.549200000	0.950400000	-0.681900000
C	-0.659200000	0.088800000	-0.897620000
C	-0.464600000	0.892800000	-0.798780000
C	-0.562300000	0.080400000	-0.783790000
C	-0.137100000	0.840800000	-0.844470000
C	-0.783100000	0.123000000	-0.940260000
C	-0.408000000	0.734800000	-0.754050000
C	-0.804400000	0.966500000	-0.987860000
C	-0.703400000	0.774800000	-0.991930000
C	-0.554500000	0.895200000	-0.901620000
C	-0.291100000	0.726300000	-0.870410000
C	-0.994300000	0.705500000	-0.862260000
C	-0.579000000	0.737900000	-0.949130000
C	-0.794900000	0.015400000	-0.454790000
C	-0.863200000	0.896500000	-0.368800000
C	-0.050600000	0.183100000	-0.331300000
C	-0.953800000	0.109500000	-0.373960000
C	-0.056500000	0.036400000	-0.282420000
C	-0.966400000	0.822800000	-0.276200000
C	-0.868800000	0.752900000	-0.318290000
S	-0.627020000	0.293730000	-0.838440000
S	-0.923330000	0.249690000	-0.439750000
N	-0.429502892	0.865868731	-0.857477418
N	-0.774634616	0.848135685	-0.414434766

Fractional coordinate of form II

H	-0.480674288	-0.514034986	0.263989554
H	-0.529888165	-0.716957749	0.274971027
H	-0.716843413	-0.785470854	0.933539945
H	-0.617862142	-0.490888128	0.598085398
H	-0.680502300	-0.039694442	0.123803718
H	-0.713804862	-0.139150952	0.242402618
H	-0.578706565	-0.801299844	0.161955144
H	-0.640315805	-0.270686640	0.232758465
H	-0.667927659	-0.343109594	0.398096984
H	-0.589281632	-0.266410670	0.484115148
H	-0.749539081	-0.080381532	0.981840908
H	-0.627712092	-1.002645613	0.168569729
H	-0.539039998	-0.065278853	0.483089411
H	-0.748691969	-0.324950292	0.754175194
H	-0.716828251	-0.281941533	0.484425471
H	-0.682724854	-0.390312557	0.004886561
H	-0.707330031	-0.479725085	0.178631065
H	-0.666065798	-0.567950169	0.141084608
H	-0.353361165	-0.731193873	0.321123838
H	-0.369850591	-0.046587524	0.217469675
H	-0.452088839	-0.647509498	0.543888302
H	-0.394105517	-0.533922283	0.481719658
C	-0.506370000	-0.273800000	0.374500000
C	-0.504480000	-0.458200000	0.313000000
C	-0.532520000	-0.572700000	0.317400000
C	-0.717190000	-0.895200000	0.834800000
C	-0.563510000	-0.508100000	0.379000000
C	-0.619920000	-0.601200000	0.500700000
C	-0.652250000	-0.861200000	0.384700000
C	-0.686570000	-0.109900000	0.247400000
C	-0.648670000	-0.710900000	0.499900000
C	-0.597890000	-0.775500000	0.261000000
C	-0.667140000	-0.293400000	0.262400000
C	-0.593980000	-0.628800000	0.379400000
C	-0.699030000	-0.004100000	0.547800000
C	-0.699980000	-0.867200000	0.678600000
C	-0.565300000	-0.323200000	0.439000000

C	-0.735050000	-0.059900000	0.862300000
C	-0.626210000	-0.890700000	0.263900000
C	-0.537270000	-0.209000000	0.438100000
C	-0.734750000	-0.195500000	0.734400000
C	-0.716840000	-0.170800000	0.580600000
C	-0.681530000	-0.441100000	0.140300000
C	-0.476580000	-0.152900000	0.367700000
C	-0.443870000	-0.910400000	0.414100000
C	-0.420440000	-0.020100000	0.323100000
C	-0.378740000	-0.783100000	0.347200000
C	-0.387610000	-0.959500000	0.288400000
C	-0.434090000	-0.732900000	0.472300000
C	-0.401800000	-0.672300000	0.438300000
S	-0.681910000	-0.645870000	0.640970000
S	-0.439510000	-0.230220000	0.266980000
N	-0.681460000	-0.974800000	0.389400000
N	-0.475340000	-0.990400000	0.438000000
H	-0.519325629	-0.485964596	0.763989498
H	-0.470111690	-0.283042673	0.774969857
H	-0.283156248	-0.214530988	0.433540103
H	-0.382137703	-0.509114844	0.098083905
H	-0.319497731	-0.960308806	0.623804983
H	-0.286195078	-0.860849151	0.742403670
H	-0.421293735	-0.198698880	0.661956724
H	-0.359684076	-0.729313103	0.732760021
H	-0.332072106	-0.656888993	0.898099593
H	-0.410718350	-0.733588664	0.984117230
H	-0.250460242	-0.919617847	0.481840933
H	-0.372287691	0.002644364	0.668569830
H	-0.460959914	-0.934720149	0.983092285
H	-0.251307808	-0.675050364	0.254175174
H	-0.283171666	-0.718057277	0.984427022
H	-0.317274874	-0.609691585	0.504886100
H	-0.292669846	-0.520275048	0.678631405
H	-0.333934048	-0.432049774	0.641083599
H	-0.646638697	-0.268806764	0.821123229
H	-0.630149493	-0.953411218	0.717471800
H	-0.547910781	-0.352491653	0.043885477

H	-0.605894340	-0.466077201	0.981722003
C	-0.493630000	-0.726200000	0.874500000
C	-0.495520000	-0.541800000	0.813000000
C	-0.467480000	-0.427300000	0.817400000
C	-0.282810000	-0.104800000	0.334800000
C	-0.436490000	-0.491900000	0.879000000
C	-0.380080000	-0.398800000	0.000700000
C	-0.347750000	-0.138800000	0.884700000
C	-0.313430000	-0.890100000	0.747400000
C	-0.351330000	-0.289100000	0.999900000
C	-0.402110000	-0.224500000	0.761000000
C	-0.332860000	-0.706600000	0.762400000
C	-0.406020000	-0.371200000	0.879400000
C	-0.300970000	-0.995900000	0.047800000
C	-0.300020000	-0.132800000	0.178600000
C	-0.434700000	-0.676800000	0.939000000
C	-0.264950000	-0.940100000	0.362300000
C	-0.373790000	-0.109300000	0.763900000
C	-0.462730000	-0.791000000	0.938100000
C	-0.265250000	-0.804500000	0.234400000
C	-0.283160000	-0.829200000	0.080600000
C	-0.318470000	-0.558900000	0.640300000
C	-0.523420000	-0.847100000	0.867700000
C	-0.556130000	-0.089600000	0.914100000
C	-0.579560000	-0.979900000	0.823100000
C	-0.621260000	-0.216900000	0.847200000
C	-0.612390000	-0.040500000	0.788400000
C	-0.565910000	-0.267100000	0.972300000
C	-0.598200000	-0.327700000	0.938300000
S	-0.318090000	-0.354130000	0.140970000
S	-0.560490000	-0.769780000	0.766980000
N	-0.318540000	-0.025200000	0.889400000
N	-0.524660000	-0.009600000	0.938000000
H	-0.980674288	-0.985965014	0.263989554
H	-0.029888165	-0.783042251	0.274971027
H	-0.216843413	-0.714529146	0.933539945
H	-0.117862142	-1.009111872	0.598085398
H	-0.180502300	-0.460305558	0.123803718

H	-0.213804862	-0.360849048	0.242402618
H	-0.078706565	-0.698700156	0.161955144
H	-0.140315805	-0.229313360	0.232758465
H	-0.167927659	-0.156890406	0.398096984
H	-0.089281632	-0.233589330	0.484115148
H	-0.249539081	-0.419618468	0.981840908
H	-0.127712092	-0.497354387	0.168569729
H	-0.039039998	-0.434721147	0.483089411
H	-0.248691969	-0.175049708	0.754175194
H	-0.216828251	-0.218058467	0.484425471
H	-0.182724854	-0.109687443	0.004886561
H	-0.207330031	-0.020274915	0.178631065
H	-0.166065798	-0.932049831	0.141084608
H	-0.853361165	-0.768806127	0.321123838
H	-0.869850591	-0.453412476	0.217469675
H	-0.952088839	-0.852490502	0.543888302
H	-0.894105517	-0.966077717	0.481719658
C	-0.006370000	-0.226200000	0.374500000
C	-0.004480000	-0.041800000	0.313000000
C	-0.032520000	-0.927300000	0.317400000
C	-0.217190000	-0.604800000	0.834800000
C	-0.063510000	-0.991900000	0.379000000
C	-0.119920000	-0.898800000	0.500700000
C	-0.152250000	-0.638800000	0.384700000
C	-0.186570000	-0.390100000	0.247400000
C	-0.148670000	-0.789100000	0.499900000
C	-0.097890000	-0.724500000	0.261000000
C	-0.167140000	-0.206600000	0.262400000
C	-0.093980000	-0.871200000	0.379400000
C	-0.199030000	-0.495900000	0.547800000
C	-0.199980000	-0.632800000	0.678600000
C	-0.065300000	-0.176800000	0.439000000
C	-0.235050000	-0.440100000	0.862300000
C	-0.126210000	-0.609300000	0.263900000
C	-0.037270000	-0.291000000	0.438100000
C	-0.234750000	-0.304500000	0.734400000
C	-0.216840000	-0.329200000	0.580600000
C	-0.181530000	-0.058900000	0.140300000

C	-0.976580000	-0.347100000	0.367700000
C	-0.943870000	-0.589600000	0.414100000
C	-0.920440000	-0.479900000	0.323100000
C	-0.878740000	-0.716900000	0.347200000
C	-0.887610000	-0.540500000	0.288400000
C	-0.934090000	-0.767100000	0.472300000
C	-0.901800000	-0.827700000	0.438300000
S	-0.181910000	-0.854130000	0.640970000
S	-0.939510000	-0.269780000	0.266980000
N	-0.181460000	-0.525200000	0.389400000
N	-0.975340000	-0.509600000	0.438000000
H	-0.019325629	-0.014035404	0.763989498
H	-0.970111690	-0.216957327	0.774969857
H	-0.783156248	-0.285469012	0.433540103
H	-0.882137703	0.009114844	0.098083905
H	-0.819497731	-0.539691194	0.623804983
H	-0.786195078	-0.639150849	0.742403670
H	-0.921293735	-0.301301120	0.661956724
H	-0.859684076	-0.770686897	0.732760021
H	-0.832072106	-0.843111007	0.898099593
H	-0.910718350	-0.766411336	0.984117230
H	-0.750460242	-0.580382153	0.481840933
H	-0.872287691	-0.502644364	0.668569830
H	-0.960959914	-0.565279851	0.983092285
H	-0.751307808	-0.824949636	0.254175174
H	-0.783171666	-0.781942723	0.984427022
H	-0.817274874	-0.890308415	0.504886100
H	-0.792669846	-0.979724952	0.678631405
H	-0.833934048	-0.067950226	0.641083599
H	-0.146638697	-0.231193236	0.821123229
H	-0.130149493	-0.546588782	0.717471800
H	-0.047910781	-0.147508347	0.043885477
H	-0.105894340	-0.033922799	0.981722003
C	-0.993630000	-0.773800000	0.874500000
C	-0.995520000	-0.958200000	0.813000000
C	-0.967480000	-0.072700000	0.817400000
C	-0.782810000	-0.395200000	0.334800000
C	-0.936490000	-0.008100000	0.879000000

C	-0.880080000	-0.101200000	0.000700000
C	-0.847750000	-0.361200000	0.884700000
C	-0.813430000	-0.609900000	0.747400000
C	-0.851330000	-0.210900000	0.999900000
C	-0.902110000	-0.275500000	0.761000000
C	-0.832860000	-0.793400000	0.762400000
C	-0.906020000	-0.128800000	0.879400000
C	-0.800970000	-0.504100000	0.047800000
C	-0.800020000	-0.367200000	0.178600000
C	-0.934700000	-0.823200000	0.939000000
C	-0.764950000	-0.559900000	0.362300000
C	-0.873790000	-0.390700000	0.763900000
C	-0.962730000	-0.709000000	0.938100000
C	-0.765250000	-0.695500000	0.234400000
C	-0.783160000	-0.670800000	0.080600000
C	-0.818470000	-0.941100000	0.640300000
C	-0.023420000	-0.652900000	0.867700000
C	-0.056130000	-0.410400000	0.914100000
C	-0.079560000	-0.520100000	0.823100000
C	-0.121260000	-0.283100000	0.847200000
C	-0.112390000	-0.459500000	0.788400000
C	-0.065910000	-0.232900000	0.972300000
C	-0.098200000	-0.172300000	0.938300000
S	-0.818090000	-0.145870000	0.140970000
S	-0.060490000	-0.730220000	0.766980000
N	-0.818540000	-0.474800000	0.889400000
N	-0.024660000	-0.490400000	0.938000000

Extracted conformation from form I

H 2.298443 3.655838 11.565455
H 2.120373 6.948455 6.429851
H 3.381942 3.504776 9.377237
H 0.641363 7.473423 10.476251
H 1.744427 7.353874 8.321382
H 4.440029 3.531746 7.672563
H 7.123610 5.709550 3.217238
H 6.992863 4.690455 4.650486
H 1.045300 7.066547 1.462298
H 5.240385 3.247682 5.410241
H 0.882353 5.520929 -0.477964
H 2.461868 3.601091 -0.651367
H 5.903651 4.000045 1.871594
H 5.707617 2.979361 3.291535
H 8.518618 3.997711 2.071719
H 8.259095 2.846431 3.401247
H 9.211979 4.311253 3.658785
H 4.160306 3.232783 1.077467
H -2.362206 7.479676 15.174805
H -2.660150 6.023568 17.173755
H -1.327213 3.947687 17.432513
H 0.331178 3.270017 15.690925
C 2.646582 5.405357 8.647196
C 2.178343 4.460737 10.842884
C 2.822884 6.140308 6.240282
C 2.795927 4.391957 9.605480
C 1.239507 6.591818 10.244185
C 1.364655 5.557894 11.176181
C 1.875658 6.522483 9.009753
C 4.101007 4.241638 6.921963
C 3.212243 5.271679 7.271718
C 2.709061 6.039357 2.340391
C 4.173902 4.952184 4.599859
C 3.307167 5.992763 4.942528
C 7.070020 4.663750 3.555392
C 1.735177 6.229057 1.365647
C 4.567478 4.075789 5.622380

C 1.649588 5.360983 0.277519
C 2.522378 4.297661 0.184479
C 3.613380 4.965496 2.248952
C 5.804555 4.028641 2.962408
C 8.328482 3.913268 3.148716
C 3.500217 4.092984 1.162881
C 0.647629 5.632221 12.463419
C -0.111736 4.972707 14.429137
C -1.792010 6.562420 15.286382
C -0.876749 6.154175 14.311181
C -1.941843 5.748704 16.403771
C -1.183263 4.563908 16.545960
C -0.262313 4.176186 15.583788
S 2.864247 7.176062 3.693237
S -0.482244 6.931781 12.807231
N 4.593601 4.802801 3.258045
N 0.739085 4.704440 13.385934

Extracted conformation from form II

H 2.298443 3.655838 11.565455
H 2.120373 6.948455 6.429851
H 3.381942 3.504776 9.377237
H 0.641363 7.473423 10.476251
H 1.744427 7.353874 8.321382
H 4.440029 3.531746 7.672563
H 7.123610 5.709550 3.217238
H 6.992863 4.690455 4.650486
H 1.045300 7.066547 1.462298
H 5.240385 3.247682 5.410241
H 0.882353 5.520929 -0.477964
H 2.461868 3.601091 -0.651367
H 5.903651 4.000045 1.871594
H 5.707617 2.979361 3.291535
H 8.518618 3.997711 2.071719
H 8.259095 2.846431 3.401247
H 9.211979 4.311253 3.658785
H 4.160306 3.232783 1.077467
H -2.362206 7.479676 15.174805
H -2.660150 6.023568 17.173755
H -1.327213 3.947687 17.432513
H 0.331178 3.270017 15.690925
C 2.646582 5.405357 8.647196
C 2.178343 4.460737 10.842884
C 2.822884 6.140308 6.240282
C 2.795927 4.391957 9.605480
C 1.239507 6.591818 10.244185
C 1.364655 5.557894 11.176181
C 1.875658 6.522483 9.009753
C 4.101007 4.241638 6.921963
C 3.212243 5.271679 7.271718
C 2.709061 6.039357 2.340391
C 4.173902 4.952184 4.599859
C 3.307167 5.992763 4.942528
C 7.070020 4.663750 3.555392
C 1.735177 6.229057 1.365647
C 4.567478 4.075789 5.622380

C 1.649588 5.360983 0.277519
C 2.522378 4.297661 0.184479
C 3.613380 4.965496 2.248952
C 5.804555 4.028641 2.962408
C 8.328482 3.913268 3.148716
C 3.500217 4.092984 1.162881
C 0.647629 5.632221 12.463419
C -0.111736 4.972707 14.429137
C -1.792010 6.562420 15.286382
C -0.876749 6.154175 14.311181
C -1.941843 5.748704 16.403771
C -1.183263 4.563908 16.545960
C -0.262313 4.176186 15.583788
S 2.864247 7.176062 3.693237
S -0.482244 6.931781 12.807231
N 4.593601 4.802801 3.258045
N 0.739085 4.704440 13.385934

***syn*-confomer optimized in vacuum condition**

H	-3.42362200	1.04699600	1.90883200
H	1.13008600	-1.57783500	-0.81837000
H	-0.96366000	1.20397100	1.94490100
H	-3.23023100	-0.87765700	-1.92393600
H	-0.79715100	-0.68966100	-1.90463400
H	0.84987700	2.33990000	0.91561400
H	5.63645100	2.29802800	-2.06690900
H	4.25483300	2.97550100	-1.23039100
H	5.68380400	-3.87789000	-0.07299300
H	3.28389800	2.49789800	0.95960700
H	7.69148000	-3.73915400	1.38393200
H	8.41659200	-1.52426400	2.25721000
H	6.76149400	1.78379900	0.03260100
H	5.47147100	2.49411900	0.98610600
H	7.12068400	4.08678300	-1.04788100
H	5.72039500	4.75595100	-0.20224900
H	5.79425800	4.80542500	-1.96537100
H	7.16421400	0.50547900	1.68274600
H	-8.91363900	-1.23191900	-1.74032200
H	-10.73154100	-0.51274000	-0.21851000
H	-10.21846200	0.67842900	1.88693500
H	-7.85661900	1.17554900	2.51234500
C	-0.68571000	0.27141900	0.02119300
C	-2.84109000	0.67126900	1.07749200
C	1.59078400	-0.66548900	-0.45811000
C	-1.45823600	0.76458200	1.08628800
C	-2.74610000	-0.42113900	-1.06773200
C	-3.51115000	0.07452700	-0.00301500
C	-1.36138500	-0.32291300	-1.05517300
C	1.43683000	1.51015200	0.53967300
C	0.79027300	0.37570100	0.03355100
C	5.34598600	-1.76175800	-0.07526400
C	3.62251100	0.58188200	0.03065700
C	2.97643800	-0.55815100	-0.47958600
C	5.33606600	2.83508200	-1.16205000
C	6.02824200	-2.91815900	0.29492500
C	2.82401500	1.60681000	0.55311500

C	7.15174800	-2.83882100	1.11593300
C	7.55841200	-1.59849800	1.59917900
C	5.75279800	-0.50174400	0.40035500
C	5.68615900	1.96087200	0.04798300
C	6.03228200	4.19644200	-1.09006700
C	6.85487000	-0.44300100	1.26328600
C	-4.97541400	-0.00888700	0.01786800
C	-7.05861700	0.25364500	0.74237000
C	-8.68510000	-0.71025900	-0.81892500
C	-7.36759300	-0.42710900	-0.45889700
C	-9.70008200	-0.30346300	0.04034800
C	-9.40750200	0.37305300	1.23599800
C	-8.09692100	0.65462500	1.59360000
S	3.97045800	-1.84722800	-1.20854800
S	-5.88451900	-0.79942200	-1.30771000
N	5.03309700	0.65132600	0.00557800
N	-5.71519800	0.46663000	0.97026500

***anti*-confomer optimized in vacuum condition**

S	3.92166700	-1.88194000	-1.22816900
N	5.03870600	0.61533700	-0.06120400
C	1.57212700	-0.66394700	-0.44218100
C	-0.68266400	0.30434400	0.07393300
C	0.79433000	0.39178700	0.05501600
C	3.62826300	0.56343100	-0.00704600
C	-1.38900000	-0.29305100	-0.98357400
C	-2.77251900	-0.37722400	-0.96979800
C	-4.97302000	0.03470600	0.09273100
C	5.75221800	-0.54250400	0.33081400
C	-3.50998100	0.13801800	0.10857200
C	1.46458600	1.52345600	0.53577300
C	2.95822200	-0.57367000	-0.49236900
C	-1.42537600	0.81511800	1.14913000
C	5.32077000	-1.80205200	-0.12371900
C	2.85283900	1.60346300	0.52029600
C	5.99629500	-2.96303000	0.24443200
C	-2.81109300	0.73547900	1.16671500
C	7.56816700	-1.64939800	1.50445300
C	5.34493000	2.78271700	-1.25804400
C	5.70827100	1.91710300	-0.04571900
C	7.13697500	-2.88934200	1.04198600
C	6.87201600	-0.48873500	1.17096300
C	6.05749600	4.13676400	-1.21361800
N	-5.65101900	-0.51010200	-0.86843600
S	-5.96872800	0.67115600	1.43893100
C	-7.39665100	0.13274100	0.58447200
C	-7.00936300	-0.47575800	-0.63263500
C	-7.99237300	-0.98668000	-1.49068400
C	-9.32630700	-0.88283800	-1.12383000
C	-9.69673300	-0.27638100	0.08771300
C	-8.73756700	0.23723300	0.95392100
H	3.33137900	2.49332600	0.90749800
H	0.89537300	2.36458100	0.91383600
H	1.09312200	-1.57371200	-0.78458000
H	5.63299900	-3.92203900	-0.10683100
H	7.67112800	-3.79352500	1.30822200

H	8.44027800	-1.57916900	2.14437300
H	7.20122100	0.45999000	1.57447100
H	-0.90976900	1.25695700	1.99372500
H	-3.34714400	1.13379600	2.02106300
H	-3.30457100	-0.83212700	-1.79539900
H	-0.84493800	-0.67439100	-1.84004200
H	-7.69219500	-1.45188300	-2.42164300
H	-10.09498400	-1.27463100	-1.77977700
H	-10.74512800	-0.20660000	0.35364800
H	-9.02605900	0.70431500	1.88769900
H	5.62199900	2.23300400	-2.16272300
H	4.26420900	2.93460000	-1.30734400
H	5.51852500	2.46280600	0.89062500
H	6.78087300	1.72669900	-0.08021800
H	5.80989300	4.73918200	-2.09080300
H	5.76860200	4.70903900	-0.32614500
H	7.14521300	4.01521000	-1.19057900

Stable state of 3,10-dimethyl-10*H*-phenothiazine

C	3.09442200	1.10573100	0.46823100
C	3.36975600	-0.25764500	0.37829300
C	2.32325200	-1.10856900	0.00453100
C	1.06308100	-0.61051800	-0.30807500
C	0.78739500	0.76559800	-0.21322800
C	1.82267200	1.61162000	0.19638500
S	-0.21067100	-1.70637400	-0.90493400
C	-1.61343500	-0.89232900	-0.16400500
C	-1.62669500	0.51355400	-0.08691100
N	-0.50470500	1.24635800	-0.53322300
C	-2.70496500	-1.63871300	0.27121700
C	-3.84945200	-1.00207300	0.75029400
C	-3.87621100	0.38659500	0.82412700
C	-2.77055200	1.13839400	0.42729500
C	-0.66450200	2.67111500	-0.77692900
C	4.74602800	-0.81063900	0.65763900
H	3.87770700	1.79265900	0.77112200
H	2.49496800	-2.17831000	-0.05840300
H	1.64680600	2.67310400	0.31021300
H	-2.66127400	-2.72061100	0.21800100
H	-4.70321100	-1.58872800	1.06760900
H	-4.75364300	0.89616000	1.20558000
H	-2.80500800	2.21534000	0.52321400
H	5.26275200	-1.07031300	-0.27256900
H	4.69430000	-1.71817900	1.26551800
H	5.36595500	-0.08477300	1.18848500
H	-0.67896300	3.27412600	0.14242400
H	0.15582100	3.01698600	-1.40640800
H	-1.59729800	2.83729400	-1.31647100

Transition state of 3,10-dimethyl-10*H*-phenothiazine

C	-3.25709300	1.11945700	0.01940600
C	-3.50733800	-0.24477000	-0.06785600
C	-2.39401700	-1.08698200	-0.05361200
C	-1.09110300	-0.60288000	0.03158100
C	-0.82607300	0.78397800	0.06437500
C	-1.95654300	1.61770200	0.08333900
S	0.16649000	-1.84622500	0.19939500
C	1.64874100	-0.88949500	0.01088600
C	1.66283700	0.52173100	0.03067600
N	0.49135500	1.30810800	0.08576200
C	2.83210700	-1.61665100	-0.09506600
C	4.07133800	-0.98403600	-0.14906100
C	4.10941800	0.40131400	-0.09549300
C	2.92872500	1.13792900	-0.01056800
C	0.69793800	2.74910800	-0.02478000
C	-4.90325700	-0.80363400	-0.18248100
H	-4.08284700	1.82271600	0.03984500
H	-2.54207600	-2.16220700	-0.09583500
H	-1.85436900	2.68810800	0.16739600
H	2.77720100	-2.69975500	-0.12629400
H	4.98004800	-1.56837400	-0.22558200
H	5.05594000	0.92868400	-0.12144200
H	3.01480000	2.21193000	0.03397500
H	-5.04760200	-1.65750900	0.48552800
H	-5.10704700	-1.14915500	-1.20173100
H	-5.65349700	-0.05020500	0.06750800
H	1.31092600	2.98165800	-0.90068000
H	-0.23960100	3.27180500	-0.14893200
H	1.19072000	3.14851200	0.86753500

References

[S1] Agilent CrysAlis PRO. Agilent Technologies Ltd, Yarnton, Oxfordshire, England, 2014.

[S2] G. M. Sheldrick, *Acta Crystallogr. Sect. A Found. Adv.* 2015, **71**, 3.

[S3] G. M. Sheldrick, *Acta Crystallogr. Sect. C Struct. Chem.* 2015, **71**, 3.

[S4] O. V. Dolomanov, L. J. Bourhis, R. J. Gildea, J. A. K. Howard, H. Puschmann, *J. Appl. Crystallogr.* 2009, **42**, 339.

[S5] C. F. Macrae, I. Sovago, S. J. Cottrell, P. T. A. Galek, P. McCabe, E. Pidcock, M. Platings, G. P. Shields, J. S. Stevens, M. Towler, P. A. Wood, *J. Appl. Crystallogr.* 2020, **53**, 226.

[S6] P. Giannozzi, S. Baroni, N. Bonini, M. Calandra, R. Car, C. Cavazzoni, D. Ceresoli, G. L. Chiarotti, M. Cococcioni, I. Dabo, A. Dal Corso, S. de Gironcoli, S. Fabris, G. Fratesi, R. Gebauer, U. Gerstmann, C. Gougoussis, A. Kokalj, M. Lazzeri, L. Martin-Samos, N. Marzari, F. Mauri, R. Mazzarello, S. Paolini, A. Pasquarello, L. Paulatto, C. Sbraccia, S. Scandolo, G. Sclauzero, A. P. Seitsonen, A. Smogunov, P. Umari, R. M. Wentzcovitch, *J. Phys. Condens. Matter* 2009, **21**, 395502.

[S7] P. Giannozzi, O. Andreussi, T. Brumme, O. Bunau, M. Buongiorno Nardelli, M. Calandra, R. Car, C. Cavazzoni, D. Ceresoli, M. Cococcioni, N. Colonna, I. Carnimeo, A. Dal Corso, S. de Gironcoli, P. Delugas, R. A. DiStasio, A. Ferretti, A. Floris, G. Fratesi, G. Fugallo, R. Gebauer, U. Gerstmann, F. Giustino, T. Gorni, J. Jia, M. Kawamura, H.-Y. Ko, A. Kokalj, E. Küçükbenli, M. Lazzeri, M. Marsili, N. Marzari, F. Mauri, N. L. Nguyen, H.-V. Nguyen, A. Otero-de-la-Roza, L. Paulatto, S. Poncé, D. Rocca, R. Sabatini, B. Santra, M. Schlipf, A. P. Seitsonen, A. Smogunov, I. Timrov, T. Thonhauser, P. Umari, N. Vast, X. Wu, S. Baroni, *J. Phys. Condens. Matter* 2017, **29**, 465901.

[S8] P. Giannozzi, O. Baseggio, P. Bonfà, D. Brunato, R. Car, I. Carnimeo, C. Cavazzoni, S. de Gironcoli, P. Delugas, F. Ferrari Ruffino, A. Ferretti, N. Marzari, I. Timrov, A. Urru, S. Baroni, *J. Chem. Phys.* 2020, **152**, 154105.

[S9] *Winmostar V11.3.1,* X-Ability Co. Ltd., Tokyo, Japan, 2022.

[S10] A. Dal Corso, *Comput. Mater. Sci.* 2014, **95**, 337.

[S11] *Gaussian 16, Revision C.01*, M. J. Frisch, G. W. Trucks, H. B. Schlegel, G. E. Scuseria, M. A. Robb, J. R. Cheeseman, G. Scalmani, V. Barone, G. A. Petersson, H. Nakatsuji, X. Li, M. Caricato, A. V. Marenich, J. Bloino, B. G. Janesko, R. Gomperts, B. Mennucci, H. P. Hratchian, J. V. Ortiz, A. F. Izmaylov, J. L. Sonnenberg, D. Williams-Young, F. Ding, F. Lipparini, F. Egidi, J. Goings, B. Peng, A. Petrone, T. Henderson, D. Ranasinghe, V. G. Zakrzewski, J. Gao, N. Rega, G. Zheng, W. Liang, M. Hada, M. Ehara, K. Toyota, R. Fukuda, J. Hasegawa, M. Ishida, T. Nakajima, Y. Honda, O. Kitao, H. Nakai, T. Vreven, K. Throssell, J. A. Montgomery, Jr., J. E. Peralta, F. Ogliaro, M. J. Bearpark, J. J. Heyd, E. N. Brothers, K. N. Kudin, V. N. Staroverov, T. A. Keith, R. Kobayashi, J. Normand, K. Raghavachari, A. P. Rendell, J. C. Burant, S. S. Iyengar, J. Tomasi, M. Cossi, J. M. Millam, M. Klene, C. Adamo, R. Cammi, J. W. Ochterski, R. L. Martin, K. Morokuma, O. Farkas, J. B. Foresman and D. J. Fox, Gaussian, Inc., Wallingford CT, 2019.
